# Novel Dual-Target Kinase Inhibitors of EGFR and ALK Were Designed, Synthesized, and Induced Cell Apoptosis in Non-Small Cell Lung Cancer

**DOI:** 10.3390/molecules28052006

**Published:** 2023-02-21

**Authors:** Yangyang Fan, Wei Li, Wenyan Nie, Han Yao, Yuanyuan Ren, Mengxuan Wang, Haoran Nie, Chenxi Gu, Jiadai Liu, Baijiao An

**Affiliations:** 1 School of Pharmacy, Binzhou Medical University, Yantai 264003, China; 2 School of Pharmaceutical Sciences, Sun Yat-sen University, Guangzhou 510006, China; 3 Shandong Technology Innovation Center of Molecular Targeting and Intelligent Diagnosis and Treatment, Yantai 264003, China

**Keywords:** non-small-cell lung cancer (NSCLC), EGFR, ALK, kinase inhibitor, therapeutic strategy

## Abstract

ALK-positive NSCLC coexisting with EGFR mutations is a frequently occurring clinical phenomenon. Targeting ALK and EGFR simultaneously may be an effective way to treat these cancer patients. In this study, we designed and synthesized ten new dual-target EGFR/ALK inhibitors. Among them, the optimal compound **9j** exhibited good activity with IC_50_ values of 0.07829 ± 0.03 μM and 0.08183 ± 0.02 μM against H1975 (EGFR ^T790M/L858R^) and H2228 (EML4-ALK) cells, respectively. Immunofluorescence assays indicated that the compound could simultaneously inhibit the expression of phosphorylated EGFR and ALK proteins. A kinase assay demonstrated that compound **9j** could inhibit both EGFR and ALK kinases; thus, exerting an antitumor effect. Additionally, compound **9j** induced apoptosis in a dose-dependent manner and inhibited the invasion and migration of tumor cells. All of these results indicate that **9j** is worthy of further study.

## 1. Introduction

Non-small-cell lung cancer (NSCLC) is one of the most deadly cancers in the world [1] and accounts for more than 350 deaths every day [2]. NSCLC is the most common subtype of lung cancer, making up 80–85% of all lung cancer cases [3,4]. The epidermal growth factor receptor (EGFR) and anaplastic lymphoma kinase (ALK) family play a significant role in cell signal transduction, and their overexpression is implicated in the pathogenesis of numerous human solid cancers, especially NSCLC [5,6].

EGFR belongs to the ErbB family and is related to cell proliferation, survival, migration, adhesion, and differentiation [7,8]. So far, small molecule kinase inhibitors (EGFR-TKIs) have reached the fourth generation. The first generation of EGFR inhibitors, such as Gefitinib, Erlotinib and Icotinib, have exhibited a great deal of activity against NSCLCs with EGFR (L858R) mutations and EGFR deletions [9,10,11]. Second-generation EGFR inhibitors afatinib [12], dacomitinib [13], and neratinib [14] have been used for the treatment of the mutation, but suffer from a narrow therapeutic dose window. The third generation of EGFR-TKIs, for example, osimertinib, overcomes the clinical side effects of the second generation [15]. However, drug resistance develops after a period of use. As a result, fourth-generation inhibitors were developed [16]. The ALK fusion gene is another important tumor driver gene found in NSCLC after mutations in the epidermal growth factor receptors [17]. ALK rearrangements have been detected in NSCLC, leading to the development of numerous ALK inhibitors to treat NSCLC that has ALK rearrangements [18]. A mere four years after the discovery of the EML4-ALK fusion protein in 2007, crizotinib [19], a first-generation small-molecule inhibitor, was approved by the FDA as an ATP-competitive tyrosine kinase inhibitor for ALK-positive NSCLC patients [20]. In clinical practice, it is considered the first-line treatment for patients with advanced NSCLC rearranged by ALK. However, drug resistance caused by multiple reasons has brought many challenges to clinical treatment [21]. Patients with metastatic NSCLC who are ALK positive or whose disease worsens after the use of crizotinib could use the second-generation ALK inhibitor, ceritinib [22].

With the development of detection technology, patients concurrent with EGFR mutation and ALK rearrangement have been found in clinical in recent years. Currently, there is no good drug in clinical treatment for patients with a co-existing ALK-EML4 and EGFR mutation [23,24], and the current solution is to use EGFR-TKI or ALK-TKI successively. However, there is no consensus on the sequence of the two inhibitors, resulting in difficulties in treating these patients [25]. Moreover, drug resistance due to EGFR bypass activation has become one of the biggest obstacles to the clinical application of ALK kinase inhibitors [26]. Therefore, the development of dual-target inhibitors with a high efficiency, low toxicity, and anti-drug resistance for dual-drive (or drug-resistant) lung cancer patients is currently of great scientific importance [27].

**Figure 1:**
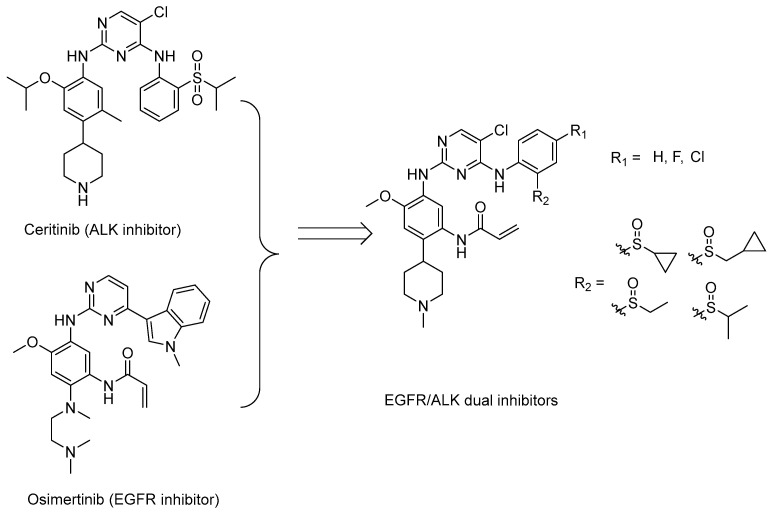
Synthesis strategy of the target compounds.

A complex multi-factor, multi-target, multi-signaling pathway collectively influences the process of single-target drug therapy during tumor development and treatment, and a multi-target drug therapy is undoubtedly an important development direction. Herein, inspired by the work of the Nathanael S. Gray Group at Harvard Medical School [28], we report our study on the design, synthesis, and evaluation of new EGFR/ALK dual-target inhibitors containing sulfoxide and a cyclopropyl group for the treatment of non-small-cell lung cancer. The design strategy of the target compound is shown in Figure 1. The aniline moiety with acrylamide group and *N*,*N*,*N*′-triethylenediamine group on the benzene ring is from the EGFR inhibitor, osimertinib; in addition, another aniline moiety with sulfoxide groups comes from the ALK inhibitor, ceritinib. Considering that halogen in the amino-parallel position of the benzene ring is beneficial to the stability of metabolism, we introduce chlorine and fluorine atoms at the R1 position to investigate their effect on the activity. In addition, as sulfoxide groups also widely exist in drugs, such as omeprazole [29], and given the special function of cyclopropyl in drugs, to study the relationship between structure and activity [30], we designed a sulfoxide group with cyclopropyl, cyclopropyl methyl, or isopropyl connected with the sulfur atom (R2) from the point of the diversity of drug molecular design.

## 2. Results and Discussion

### 2.1. Chemistry

**Scheme 1:**
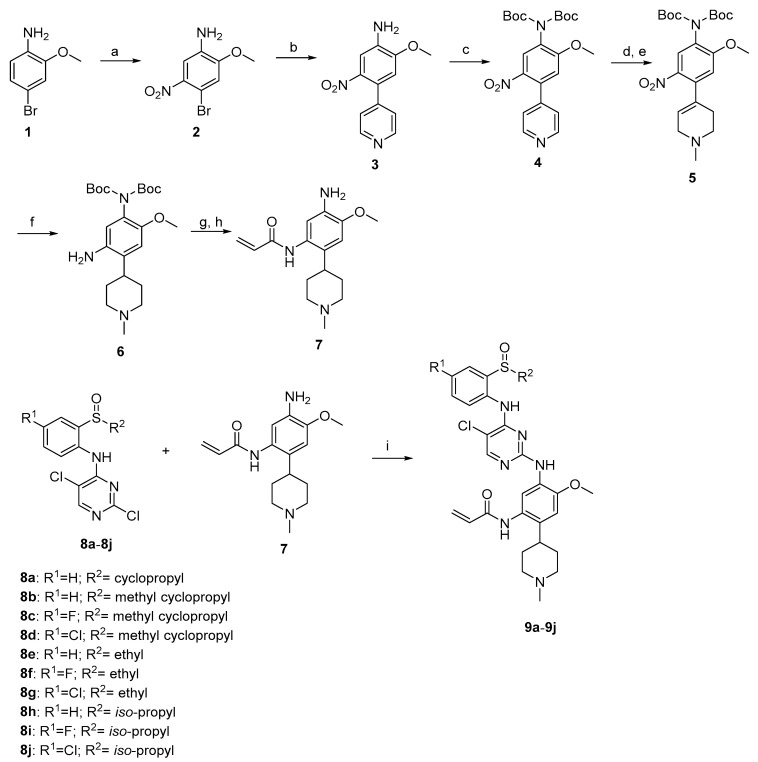
Synthetic route of compunds **9a**–**9j**. Reagents and conditions: (a) Conc.H_2_SO_4_, KNO_3_, 0 °C; (b) 4-pyidineboronic acid, dioxane, H_2_O, Na_2_CO_3_, PdCl_2_(pph_3_)_2_, reflux; (c) Et_3_N, (Boc)_2_O, DMAP, CH_2_Cl_2_, rt; (d) CH_3_I, acetonitrile, 50 °C; (e) NaBH_4_, MeOH, 0 °C; (f) Pd/C, H_2_, MeOH, 50 psi, 50 °C; (g) Acryloyl chloride, CH_2_Cl_2_,DIPEA, 0 °C; (h) TFA, CH_2_Cl_2_, rt; (i) TFA, CH_3_(CH_2_)_2_CH_2_OH, 80 °C.

The synthetic route of target compounds **9a**–**9j** is shown in Scheme 1. Nitration of 4-bromo-2-methoxyaniline using KNO_3_ in H_2_SO_4_ yielded the corresponding nitro derivatives **2**. The Suzuki coupling of **2** with 4-pyridineboronic acid afforded **3**. Compound **3** reacted with (Boc)_2_O in the presence of 4-dimethylaminopyridine to form intermediate **4**. *N*-methylation of **4,** followed by the reduction with NaBH_4_, generated **5**. By the hydrogenation of compound **5** in the presence of Pd/C, the carbon double bond and the nitro group were reduced simultaneously to obtain intermediate **6**. Reaction with acryloyl chloride, deprotection of **6** gave the key intermediate **7**, which reacted with the corresponding **8a**–**8j** to obtain the target compounds **9a**–**9j**.

### 2.2. Antiproliferative Activity against Human Lung Cancer Cells

On the basis of the synthesis, we used a CCK8 assay to screen the antiproliferative activity of the target compound in three cancer cell lines (H1975, EGFR ^T790M/L858R^, H2228, EML4-ALK, and H522 cells), using osimertinib and brigatinib as references. The results are shown in Table 1. The in vitro biological activity results showed that most of the target compounds had a high inhibitory activity against tumor cells. Among them, compounds **9b**, **9h,** and **9j** showed a good and relatively balanced antiproliferative activity for both the H1975 and H2228 cancer cell lines with IC_50_ values of 0.0689 to 0.0782 μM against the H1975 cells, and 0.076 to 0.098 μM for the H2228 cells. Further analysis showed that the structure change of the compounds had a great impact on the activity. Compound **9a**, cyclopropyl linked with a sulfoxide sulfur atom, showed a moderated activity with IC_50_ 0.4511 ± 0.07 μM against the H1975 cancer lines and 0.1214 ± 0.09 μM against H2228. It is interesting that the replacement of the R^2^ group of **9a** with methyl to obtain **9b** allowed the activity to significantly improve (0.0689μM against the H1975 cancer lines and 0.0989 μM against the H2228 cells). Comparing the activity of **9b** with that of **9c** and **9d**, it can be seen that the R^1^ group also has a greater impact on the activity. Fluorine or chlorine atoms at the position of R^1^ are not conducive to an improvement in activity (**9c**, R^1^ = F, 0.2707 μM against H1975 and 0.1027 μM against H2228; **9d**, R^1^ = Cl, 0.1143 μM against H1975 and 0.089 μM against H2228). Compounds **9e**–**9g** are ethyl linked with sulfoxide sulfur atoms and also exhibit a moderate to good antiproliferative activity with IC_50_ values ranging from 0.06901 to 0.5495 μM against the H1975 cancer lines, and from 0.0928 to 0.1037 μM against H2228. In this series, when the R^1^ group is a chlorine atom, there is no adverse effect on the activity. For example, **9g** exhibited IC_50_ values of 0.06901 μM against H1975 and 0.1037 μM against H2228 cells. The optimal antiproliferative activities (IC_50_ values of 0.07829 ± 0.03 and 0.08183 ± 0.02 μM against H1975 and H2228 cancer cell lines, respectively) were provided by compound **9j**, which features an iso-propyl as the R^2^ group and a Cl atom as the R^1^ position. Based on the above results, we can see that most of these target compounds could inhibit the proliferation of EGFR double mutant H1975 and H2228 cells effectively. Among them, compounds **9b**, **9h**, and **9j** exhibited good and comparable activities. Considering cyclopropyl ring addresses multiple roadblocks that can occur during drug discovery, such as enhancing potency, reducing off-target effects, increasing metabolic stability, and so on [30], we chose **9j** for the further study.

### 2.3. Safety Verification of Compound ***9j***

It is critical to evaluate the balance between the safety and efficacy of drugs for the development of new drugs; thus, we then conducted a cytotoxicity test on four types of nontumorigenic cell lines from different origins, including LO2 (human normal liver cells), HK2 (human kidney proximal convoluted tubule epithelial cells), HLF (human embryonic lung fibroblast), 293A (human embryonic kidney cells), and HUVEC (human umbilical vein endothelial cells). The results summarized in Table 2 showed that compound **9j** exhibited very little apparent toxicity toward the five nontumorigenic cell lines (LO2, HK2, BJ, and HLF) and a good selectivity ratio range from 255.49 to 357.31, compared with 419.51 to 1058.5 for osimertinib. In addition, we evaluated the compound’s safety in vivo by performing acute toxicity tests in mice, and **9j** was injected intravenously at concentrations of 100 mg/kg, 150 mg/kg, and 300 mg/kg. In these three doses, no mouse death occurred, and HE staining of lung and heart tissues did not indicate toxicity either (Figure S1). According to the results, we believe that the compound appears to be safe.

### 2.4. Compound ***9j***’s Activity on the EGFR and ALK Kinases

**Figure 2:**
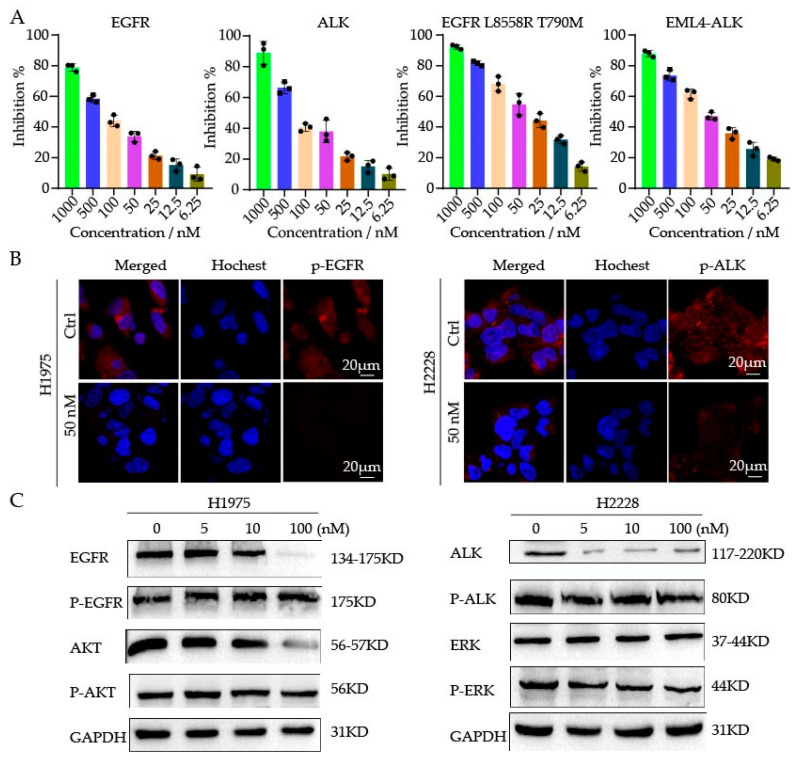
(**A**) The activity of compound **9j** against four kinases (EGFR, ALK, EGFR ^L858R T790M^, and EML4-ALK). (**B**) H1975 and H2228 cells were treated with **9j** for 2–4 h; the staining for phosphorylated EGFR (p-EGFR) and phosphorylated ALK (p-ALK) was shown as red fluorescence. (**C**) WB analysis of phosphorylated proteins in drug-treated H1975 and H2228 cells.

As compound **9j** exhibited better antitumor properties in the cell experiments, we determined to study its kinase inhibitory activity. Four kinases (EGFR, EGFR^L858R/T790M^, ALK, and EML4-ALK) were tested using an ELISA assay. As shown in Figure 2A, the IC_50_ for **9j** against EGFR^L858R/T790M^ and EML4-ALK was 35.7 ± 0.9 nM and 56.3 ± 0.3 nM, respectively. There was no effect on the wild-type EGFR or ALK kinases triggered by the compound. Consequently, the selection of the compound was good as both compounds balanced IC_50_ against EGFR and ALK, respectively. In addition, the IC_50_ values for the EGFR and ALK wild-type kinases were 236.16 ± 27 nM and 129.81 ± 15 nM, respectively, indicating a good selectivity and low toxicity. Following that, fluorescence assays were used to measure the phosphorylation of the EGFR and ALK protein affect with compound **9j** at the cellular level. As shown in Figure 2B, the expression levels of the phosphorylated EGFR and ALK proteins decreased significantly after the compound **9j** treatment for 2 to 4 h. Next, WB assay was used to test the protein expression of H1975 and H2228 cells at the concentration of 5, 10, and 100 nM. The results showed that the expression of p-EGFR and its downstream protein p-AKT, p-ALK, and its downstream protein p-ERK were inhibited after 3 h treatment of the compound (Figure 2C). These results indicated that the compounds could not only inhibit the activity of the EGFR and ALK kinases, but also inhibit the expression of the p-EGFR, p-ALK, p-AKT, and p-ERK; thus, exerting antitumor effects.

### 2.5. Moleculardocking

**Figure 3:**
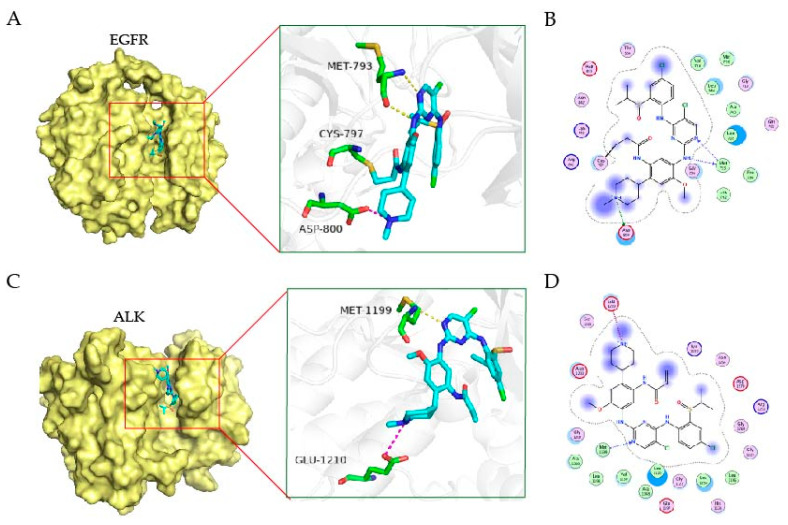
The binding mode of EGFR and ALK kinase with the compound. The 3D binding mode of EGFR kinase (**A**) and ALK (**C**) with the compound **9j**. The compound is colored in cyan. The 2D binding mode of EGFR kinase (**B**) and ALK (**D**) with the compound **9j**.

To study the possible interaction between EGFR or ALK kinase with the target compounds, we performed the docking study with the crystal structure of kinase as templates (EGFR, PDB:3IKA, ALK, PDB, 2XB7) [31,32] and conducted to rationalize the inhibitory activity using Schrödinger suites. The binding mode between EGFR kinase and the compound **9j** was shown in Figure 3A,B; like most kinase inhibitors, **9j** occupies the ATP-binding pocket of the kinase, where isopropyl linked sulfoxide deep into the pocket and *N*-methylpiperidine to the solvent. Similar to Osimertinib, **9j** exerts its inhibitory effect by forming covalent bonds with the Cys-797 site. In addition, **9j** and Met-793 form two hydrogen bonds in the hinge region. *N*-methylpiperidine formed a salt bridge with Asp-800. These interaction sites facilitate the binding of **9j** to EGFR kinase. In Figure 3C,D, compound **9j** is the same as brigatinib, located near the hinge region of the ATP-binding pocket of the kinase; it forms a hydrogen bond to nitrogen on Met1199 through 2-aminopyrimidine fragments. *N*-methylpiperidine of **9j** extends outward and forms a salt bond with Glu1210. These interactions suggest why **9j** has an inhibitory effect on ALK.

### 2.6. Apoptosis Assay

**Figure 4:**
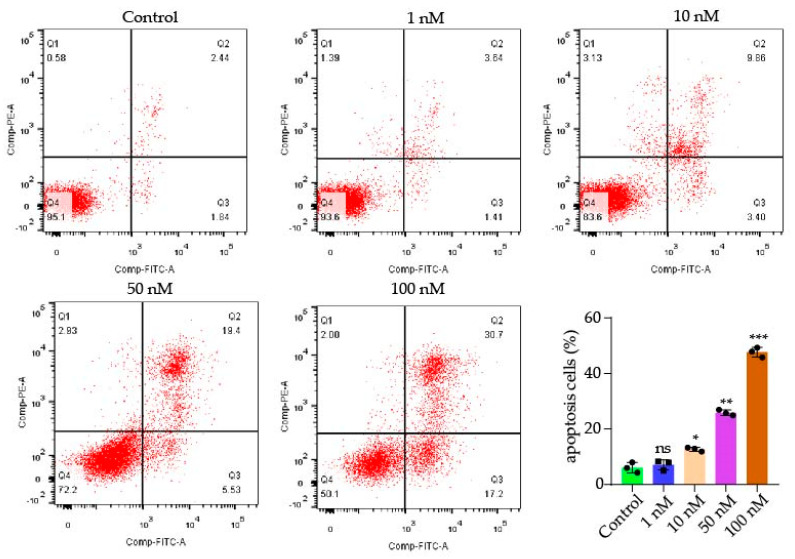
Dot plot representation of annexin-V-FITC fluorescence (x-axis) vs. PI fluorescence (y-axis) of the apoptotic H1975 (annexin-V positive) cells. The total apoptosis cell percentages were obtained using EXPO32 ADC analysis software. *p*-values of *p* < 0.05 (*), *p* < 0.01 (**), *p* < 0.001 (***). ns stands for not statistically significant.

The death of cells occurs by apoptosis, and the uncontrolled division and multiplication of cells causes cancer. Inhibiting cellular apoptosis is primarily responsible for cancer cell survival, since cellular apoptosis is negatively correlated with cell growth. We then verified the antitumor effect of compound **9j** by inducing apoptosis through flow cytometry. As shown in Figure 4, the number of apoptotic cells gradually increased with the increase of the administration concentration. Compared with the control at 6.07%, the apoptotic rates of compound **9j** at 10 nM, 50 nM, and 100 nM for 48 h were 12.7%, 26.8%, and 47.67%, respectively.

### 2.7. Compound ***9j*** Inhibited the Invasion and Migration of TUMOR Cells

**Figure 5:**
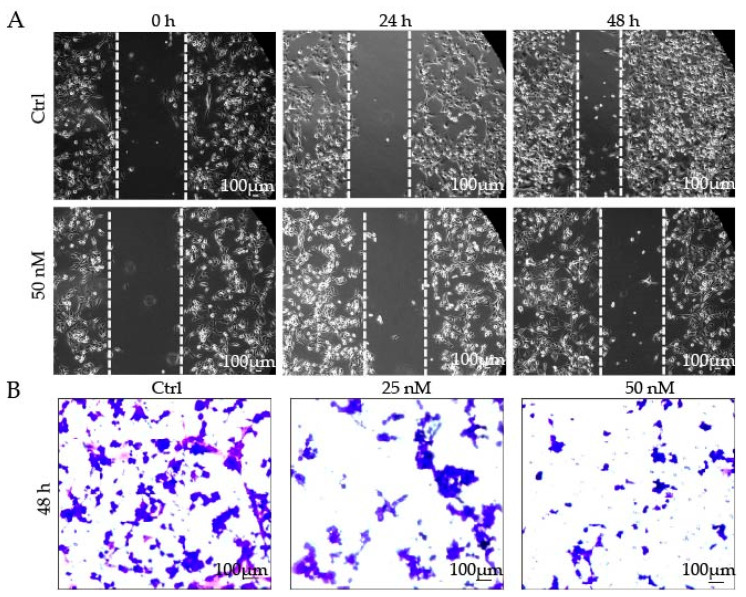
(**A**) Compound **9j** inhibited the migration of H1975 cells; (**B**) H1975 cell invasion was inhibited by compound **9j**.

The migration and invasion of tumor cells directly contribute to metastasis. Metastasis is the leading cause of death in cancer patients [33]. To determine the ability of **9j** to inhibit tumor cell migration, the cell scratching assay was performed. Following scratching, phase contrast pictures were taken of the scratched and healing cell monolayers. As shown in Figure 5A, the scratch in the PBS group had a healing rate of about 70% after 48 h, and the healing rate clearly decreased following treatment with **9j**. Further analysis of the transwell assay (Figure 5B) revealed that **9j** inhibited the H1975 cell proliferation and reduced its invasion ability in a concentration-dependent manner. All of these results indicate that **9j** has a good ability to inhibit cell migration and invasion.

## 3. Materials and Methods

### 3.1. Chemistry General Methods

All the reagents used in the synthesis were obtained commercially and used without further purification, unless otherwise specified. The ^1^H NMR and ^13^C NMR spectra were recorded using TMS as the internal standard on a Bruker BioSpin GmbH spectrometer at 400 and 100 MHz, respectively. High-resolution mass spectra (HR-MS) were obtained using a Shimadzu LCMS-ITTOF mass spectrometer. The reactions were monitored by thinlayer chromatography (TLC) on glass-packed precoated silica gel plates, and visualized in an iodine chamber or with a UV lamp. Flash column chromatography was performed using silica gel (200–300 mesh) purchased from Qingdao Haiyang Chemical Co., Ltd. Chemical and solvents were of reagent grade and used as obtained from Alfa Aesar (Ward Hill, MA, USA) and Sigma-Aldrich (St. Louis, MO, USA) without further purification. The purity of all the final synthesised compounds were ≥95% as determined by high-performance liquid chromatography (HPLC) with a TC-C18 column (4.6 × 250 mm, 5 μm).

#### 3.1.1. The Synthesis of the Target Compounds

##### 4-Bromo-2-methoxy-5-nitroaniline (**2**)

To a solution of KNO_3_ (217 mg, 2.3 mmol) in conc.H_2_SO_4_ (3.5 mL) at 0 °C for 10 min, compound **1** (400 mg, 2.0 mmol) was added. After the addition was completed, the mixture was stirred violently for 5 min at 0 °C. The reaction mixture was quenched with ice and basified with 6 N NaOH (aq) solution (pH 8). The resulting suspension was filtered through a Buchner funnel and washed with water to give **2** as a yellow solid (355 mg, 72%). ^1^H NMR (400 MHz, CDCl_3_) *δ*: 7.35 (s, 1H), 6.99 (s, 1H), 3.93 (s, 3H). ^1^H NMR Spectrum of **2** in CDCl_3_ is shown in Figure S2.

##### 2-Methoxy-5-nitro-4-(pyridin-4-yl)aniline (**3**)

4-Pyridineboronic acid (2.5 g, 20 mmol) and **2** (4.9 g, 20 mmol) were dissolved in mixture of dioxane (50.0 mL) and 2 N Na_2_CO_3_ (aq) solution (25.0 mL). Palladium (II)bis(triphenylphosphine) dichloride (330 mg, 0.47 mmol) was added. Then, the reaction mixture was stirred at 101 °C under nitrogen for 6 h, followed by cooling to room temperature. The mixture was concentrated under reduced pressure. The residue was purified by flash column chromatography on silica gel to give **3** as a yellow solid (3.9 g, 79%). ^1^H NMR (400 MHz, CDCl_3_) *δ*: 8.62 (d, *J* = 5.7 Hz, 2H), 7.39 (s, 1H), 7.19 (dd, *J* = 4.5, 1.6 Hz, 2H), 6.62 (s, 1H), 4.22 (s, 2H), 3.93 (s, 3H). ^1^H NMR Spectrum of **3** in CDCl_3_ is shown in Figure S3.

##### *N*, *N*-Ditert-butyl (2-methoxy-5-nitro-4-(pyridin-4-yl) phenyl) Carbamate (**4**)

Compound **3** (282.6 mg, 1.2 mmol) was dissolved in DCM (5.0 mL). TEA (0.3 mL, 2.3 mmol) and 4-dimethylaminopyridine (14.1 mg, 0.12 mmol) were added, followed by (Boc)_2_O (0.67 mL, 2.9 mmol). The mixture was stirred at room temperature for overnight and then, concentrated under vacuum. The residue was purified by flash column chromatography on silica gel to give **4** as a light yellow solid (333.9 mg, 65%). ^1^H NMR (400 MHz, CDCl_3_) *δ*: 8.69 (d, *J* = 5.6 Hz, 2H), 7.99 (s, 1H), 7.25 (d, *J* = 5.4 Hz, 2H), 6.77 (s, 1H), 3.93 (s, 3H), 1.48 (s, 18H). ^1^H NMR Spectrum of **4** in CDCl_3_ is shown in Figure S4.

##### *N*, *N*-Ditert-butyl(2-methoxy-4-(1-methyl-1,2,3,6-tetrahydropyridin-4-yl)-5nitrophenyl) Carbamate (**5**)

To a solution of **4** (317.3 mg, 0.7 mmol), acetonitrile (5.0 mL) and iodomethane (0.2 mL, 2.5 mmol) were added. The mixture was stirred at 50 °C for 4 h. The mixture was concentrated under reduced pressure. The crude product was dissolved in MeOH (5.0 mL) and cooled to 0 °C. NaBH_4_ (380.1 mg, 10.0 mmol) was slowly added. After the addition was complete, the cooling bath was removed and the mixture was stirred at room temperature for 1 h. The reaction was quenched by the slow addition of 1 N aqueous HCl. The MeOH was partially removed by vacuum, and the resulting residue was partitioned between EA and 1 N aqueous NaOH. Additional 1 N aqueous NaOH was added until the aqueous layer pH was >12. The mixture was extracted with EA and washed twice with 1 N aqueous NaOH. The organic layers were washed with brine, then dried over Na_2_SO_4_; subsequently, after filtration, the solvent was concentrated under reduced pressure and the residue was purified by flash column chromatography on silica gel to give **5** as a light-yellow solid (310.1 mg, 64%). ^1^H NMR (400 MHz, CDCl_3_) *δ*: 7.91 (s, 1H), 6.77 (s, 1H), 5.60 (s, 1H), 3.88 (s, 3H), 3.13 (s, 2H), 2.74 (d, *J* = 5.2 Hz, 2H), 2.47~2.39 (m, 5H), 1.45 (s, 18H). ^1^H NMR Spectrum of **5** in CDCl_3_ is shown in Figure S5.

##### *N*, *N*-Ditert-butyl (5-amino-2-methoxy-4-(1-methylpiperidin-4-yl) phenyl) Carbamate (**6**)

To a solution of **5** (0.5 g, 1.1 mmol), MeOH (10.0 mL) and 10% Pd/C (50 mg, 0.1 mmol) were added. The mixture was placed under 50 psi of H_2_ gas in a Parr shaker for 24 h. The reaction mixture was filtered and the filtrate concentrated under vacuum. The residue was purified by flash column chromatography on silica gel to give **6** as a white solid (431 mg, 92%). ^1^H NMR (400 MHz, CDCl_3_) *δ*: 6.73 (s, 1H), 6.49 (s, 1H), 3.72 (s, 3H), 3.02 (d, *J* = 11.5 Hz, 2H), 2.52~2.45 (m, 1H), 2.34 (s, 3H), 2.10 (td, *J* = 11.2, 4.0 Hz, 2H), 1.85~1.80 (m, 4H), 1.44 (s, 18H). ^1^H NMR Spectrum of **6** in CDCl_3_ is shown in Figure S6.

##### *N*-(5-Amino-4-methoxy-2-(1-methylpiperidin-4-yl) phenyl) Acrylamide (**7**)

To a solution of **6** (974 mg, 2.2 mmol) in DCM (15.0 mL) and TEA (0.6 mL, 4.5 mmol), acryloyl chloride was added dropwise (244.4 mg, 2.7 mmol) at 0 °C. After stirring at 0 °C for 1 h. The reaction mixture was quenched with saturated sodium bicarbonate solution and extracted with DCM. The combined organic layers were washed with brine, then dried over Na_2_SO_4_; in addition, after filtration, the solvent was concentrated under reduced pressure. The crude product (2.0 g, 4.1 mmol) was dissolved in DCM (5.0 mL). TFA (4.6 mL, 61.4 mmol) was added, and the mixture was stirred at room temperature overnight. The reaction mixture was basified with 6 N NaOH (aq) solution (pH 12) and extracted with DCM. The combined organic layers were washed with brine, then dried over Na_2_SO_4_; subsequently, after filtration, the solvent was concentrated under reduced pressure and the residue was purified by flash column chromatography on silica gel to give **7** as a white solid (997 mg, 83%). ^1^H NMR (400 MHz, MeOD) *δ*: 6.78 (s, 1H), 6.65 (s, 1H), 6.48 (dd, *J* = 17.0, 10.2 Hz, 1H), 6.34 (dd, *J* = 17.0, 1.8 Hz, 1H), 5.79 (dd, *J* = 10.2, 1.8 Hz, 1H), 3.88 (s, 3H), 3.05 (d, *J* = 11.9 Hz, 2H), 2.70~2.60 (m, 1H), 2.38 (s, 3H), 2.21 (td, *J* = 11.3, 4.8 Hz, 2H), 1.82~1.75 (m, 4H). ^1^H NMR Spectrum of **7** in CDCl_3_ is shown in Figure S7.

Compounds **8a**–**8d** were prepared according to literature [34].

Compounds **8e**–**8j** were prepared according to literature [35].

#### 3.1.2. General Procedure for Preparation of Compounds (**9a**–**9j**)

To a solution of **8a**–**8j** (1.1 mmol) and **7** (150 mg, 0.5 mmol) in 1-butanol (5.0 mL), TFA (0.5 mL) was added. The mixture was heated at 80 °C for 48 h and concentrated under reduced pressure. The residue was purified by flash column chromatography on silica gel to give **9a**–**9j**.

##### *N*-(5-((5-Chloro-4-((2-(cyclopropylsulfinyl)phenyl)amino)pyrimidin-2-yl)amino)-4-methoxy-2-(1-methylpiperidin-4-yl)phenyl)acrylamide (**9a**)

White solid, yield: 68%. ^1^H NMR (400 MHz, CDCl_3_) *δ*: 9.71 (s, 1H), 8.24 (d, *J* = 7.8 Hz, 2H), 8.06 (s, 1H), 8.01 (s, 1H), 7.51~7.44 (m, 2H), 7.35 (d, *J* = 7.5 Hz, 1H), 7.08 (t, *J* = 7.5 Hz, 1H), 6.80 (d, *J* = 13.2 Hz, 1H), 6.41 (dt, *J* = 40.0, 13.4 Hz, 2H), 5.71 (d, *J* = 10.1 Hz, 1H), 3.81 (s, 3H), 3.26 (s, 2H), 2.75 (t, *J* = 11.8 Hz, 1H), 2.64~2.49 (m, 6H), 2.18 (d, *J* = 12.0 Hz, 2H), 1.89 (dd, *J* = 31.5, 13.4 Hz, 2H), 1.19 (s, 1H), 1.06~1.00 (m, 1H), 0.90~0.81 (m, 2H). ^13^C NMR (100 MHz, CDCl3) *δ*: 165.0, 157.0, 155.7, 154.6, 147.0, 139.1, 133.4, 132.1, 131.1, 130.5, 127.7, 127.1, 126.6, 126.3, 124.1, 123.5, 116.5, 107.9, 106.2, 56.0, 55.5, 44.1, 34.6, 31.7, 30.1, 4.7, 3.6. [M + H]^+^ calculated for C_29_H_33_N_6_O_3_SCl, 581.2096; found, 581.2093. Purity: 99.6% (by HPLC). ^1^H NMR, ^3^C NMR, HRMS, and HPLC of **9a** are shown in Figures S8, S9, S28 and S38, respectively.

##### *N*-(5-((5-Chloro-4-((2-((cyclopropylmethyl)sulfinyl)phenyl)amino)pyrimidin-2-yl)amino)-4-methoxy-2-(1-methylpiperidin-4-yl)phenyl)acrylamide (**9b**)

White solid, yield: 48%. ^1^H NMR (400 MHz, CDCl_3_) *δ*: 9.89 (s, 1H), 8.31 (d, *J* = 8.2 Hz, 1H), 8.10 (s, 1H), 8.02 (s, 1H), 7.86 (s, 1H), 7.50~7.43 (m, 2H), 7.36 (d, *J* = 7.5 Hz, 1H), 7.05 (t, *J* = 7.4 Hz, 1H), 6.78 (s, 1H), 6.33 (t, *J* = 11.3 Hz, 2H), 5.70 (d, *J* = 8.3 Hz, 1H), 3.82 (s, 3H), 3.14~3.03 (m, 3H), 2.89 (dd, *J* = 12.9, 7.5 Hz, 1H), 2.65 (t, *J* = 11.6 Hz, 1H), 2.43 (s, 3H), 2.29 (s, 2H), 1.98 (d, *J* = 11.2 Hz, 2H), 1.83 (dd, *J* = 26.6, 12.7 Hz, 2H), 0.88~0.82 (m, 1H), 0.60~0.49 (m, 2H), 0.29~0.12 (m, 2H). ^13^C NMR (100 MHz, CDCl_3_) *δ*: 164.8, 157.0, 155.5, 154.7, 147.0, 139.9, 134.4, 132.1, 131.1, 128.3, 127.5, 126.9, 126.8, 126.2, 123.7, 123.2, 116.6, 107.9, 106.2, 59.1, 55.8, 45.0, 35.3, 31.4, 31.3, 5.1, 4.9, 4.8. [M + H]^+^ calculated for C_30_H_35_N_6_O_3_SCl, 595.2253; found, 595.2264. Purity: 98.3% (by HPLC). ^1^H NMR, ^3^C NMR, HRMS, and HPLC of **9b** are shown in Figures S10, S11, S29 and S39, respectively.

##### *N*-(5-((5-Chloro-4-((2-((cyclopropylmethyl)sulfinyl)-4-fluorophenyl)amino)pyrimidin-2-yl)amino)-4-methoxy-2-(1-methylpiperidin-4-yl)phenyl)acrylamide (**9c**)

White solid, yield: 56%. ^1^H NMR (400 MHz, CDCl_3_) *δ*: 9.31 (s, 1H), 8.15 (s, 1H), 8.03 (d, *J* = 16.3 Hz, 2H), 7.94 (s, 1H), 7.48 (s, 1H), 7.17 (t, *J* = 8.3 Hz, 2H), 6.77 (s, 1H), 6.43~6.27 (m, 2H), 5.72 (d, *J* = 9.4 Hz, 1H), 3.81 (s, 3H), 3.07 (d, *J* = 19.6 Hz, 2H), 3.03~2.96 (m, 1H), 2.87 (dd, *J* = 13.0, 7.6 Hz, 1H), 2.66 (t, *J* = 11.6 Hz, 1H), 2.40 (d, *J* = 22.3 Hz, 3H), 2.30 (s, 2H), 1.97 (d, *J* = 8.0 Hz, 2H), 1.82 (dd, *J* = 27.9, 12.9 Hz, 2H), 0.86 (dd, *J* = 11.1, 6.4 Hz, 1H), 0.61~0.51 (m, 2H), 0.29~0.13 (m, 2H). ^13^C NMR (100 MHz, CDCl_3_) *δ*: 164.9, 159.5, 157.1, 157.0, 155.6, 154.7, 146.9, 135.2, 134.4, 130.8, 127.3 (d, *J* = 11.5 Hz), 126.2, 119.1, 118.9, 116.5, 113.5, 113.3, 107.9, 105.7, 59.5, 55.8, 45.0, 35.3, 31.4, 31.2, 5.2, 4.9, 4.8. [M + H]^+^ calculated for C_30_H_34_N_6_O_3_FSCl, 613.2158; found, 631.2149. Purity: 98.2% (by HPLC). ^1^H NMR, ^3^C NMR, HRMS, and HPLC of **9c** are shown in Figures S12, S13, S30, and S40, respectively.

##### *N*-(5-((5-Chloro-4-((4-chloro-2-((cyclopropylmethyl)sulfinyl)phenyl)amino)pyrimidin-2-yl)amino)-4-methoxy-2-(1-methylpiperidin-4-yl)phenyl)acrylamide (**9d**)

White solid, yield: 74%. ^1^H NMR (400 MHz, CDCl_3_) *δ*: 9.84 (s, 1H), 8.48 (s, 1H), 8.32 (d, *J* = 8.8 Hz, 1H), 8.03 (d, *J* = 16.8 Hz, 2H), 7.52~7.42 (m, 2H), 7.32 (s, 1H), 6.80 (s, 1H), 6.53~6.32 (m, 2H), 5.72 (d, *J* = 10.1 Hz, 1H), 3.81 (s, 3H), 3.36 (d, *J* = 10.1 Hz, 2H), 3.09 (dd, *J* = 13.1, 7.2 Hz, 1H), 2.96~2.81 (m, 2H), 2.70 (s, 2H), 2.63 (s, 3H), 2.34~2.21 (m, 2H), 2.01~1.86 (m, 2H), 0.88~0.84 (m, 1H), 0.63~0.50 (m, 2H), 0.31~0.15 (m, 2H). ^13^C NMR (100 MHz, CDCl_3_) *δ*: 165.1, 156.9, 155.3, 154.8, 147.0, 138.5, 133.1, 132.1, 130.8, 129.4, 128.2, 127.6, 127.4, 126.4, 126.3, 124.5, 116.6, 107.9, 106.3, 59.3, 56.0, 55.4, 45.8, 43.7, 34.3, 8.5, 5.2, 4.9. [M + H]^+^ calculated for C_30_H_34_N_6_O_3_SCl_2_, 629.1863; found, 629.1866. Purity: 98.5% (by HPLC). ^1^H NMR, ^3^C NMR, HRMS, and HPLC of **9d** are shown in Figures S14, S15, S31 and S41, respectively.

##### *N*-(5-((5-Chloro-4-((2-(ethylsulfinyl)phenyl)amino)pyrimidin-2-yl)amino)-4-methoxy-2-(1-methylpiperidin-4-yl)phenyl)acrylamide (**9e**)

White solid, yield: 50%. ^1^H NMR (400 MHz, CDCl_3_) *δ*: 9.77 (s, 1H), 8.28 (d, *J* = 8.1 Hz, 1H), 8.08 (s, 1H), 8.01 (d, *J* = 3.5 Hz, 1H), 7.51~7.44 (m, 2H), 7.33 (s, 1H), 7.07 (d, *J* = 4.2 Hz, 1H), 6.80 (s, 1H), 6.40 (dd, *J* = 21.6, 11.6 Hz, 2H), 5.72 (s, 1H), 3.82 (d, *J* = 3.3 Hz, 3H), 3.24 (s, 2H), 3.10~2.99 (m, 2H), 2.73 (t, *J* = 11.7 Hz, 1H), 2.54 (s, 3H), 2.47 (s, 2H), 2.15 (s, 2H), 1.89 (dd, *J* = 32.1, 12.8 Hz, 2H), 1.22 (m, 3H). ^13^C NMR (100 MHz, CDCl_3_) *δ*: 164.9, 157.0, 155.6, 154.7, 147.0, 139.7, 133.6, 132.2, 131.1, 127.6, 126.9, 126.8, 126.3, 126.2, 123.9, 123.4, 116.6, 107.9, 106.2, 55.9, 55.6, 47.4, 44.3, 30.6, 29.6, 7.4. [M + H]^+^ calculated for C_28_H_33_N_6_O_3_SCl, 569.2096; found, 569.2096. Purity: 96.3% (by HPLC). ^1^H NMR, ^3^C NMR, HRMS, and HPLC of **9e** are shown in Figures S16, S17, S32 and S42, respectively.

##### *N*-(5-((5-Chloro-4-((2-(ethylsulfinyl)-4-fluorophenyl)amino)pyrimidin-2-yl)amino)-4-methoxy-2-(1-methylpiperidin-4-yl)phenyl)acrylamide (**9f**)

White solid, yield: 83%. ^1^H NMR (400 MHz, CDCl_3_) *δ*: 9.15 (s, 1H), 8.10 (s, 2H), 8.00 (d, *J* = 6.2 Hz, 2H), 7.49 (s, 1H), 7.18~7.13 (m, 2H), 6.76 (s, 1H), 6.42~6.30 (m, 2H), 5.73 (d, *J* = 9.1 Hz, 1H), 3.80 (s, 3H), 3.15 (s, 2H), 3.00~2.96 (m, 2H), 2.66 (t, *J* = 11.7 Hz, 1H), 2.47 (s, 3H), 2.38 (d, *J* = 22.0 Hz, 2H), 1.99 (d, *J* = 6.7 Hz, 2H), 1.83 (dd, *J* = 31.1, 13.0 Hz, 2H), 1.19 (d, *J* = 7.4 Hz, 3H). ^13^C NMR (100 MHz, CDCl_3_) *δ*: 164.9, 159.6, 157.1 (d, *J* = 4.1 Hz), 155.7, 154.7, 146.9, 134.8, 134.1, 130.8, 127.3 (d, *J* = 12.9 Hz), 126.5 (d, *J* = 6.8 Hz), 126.2, 119.0, 118.9, 116.4, 113.5, 113.3, 107.8, 105.7, 55.8, 55.7, 47.5, 44.8, 35.1, 29.6, 7.0. [M + H]^+^ calculated for C_28_H_32_N_6_O_3_FSCl, 587.2002; found, 587.2007. Purity: 96.5% (by HPLC). ^1^H NMR, ^3^C NMR, HRMS, and HPLC of **9f** are shown in Figures S18, S19, S33 and S43, respectively.

##### *N*-(5-((5-Chloro-4-((4-chloro-2-(ethylsulfinyl)phenyl)amino)pyrimidin-2-yl)amino)-4-methoxy-2-(1-methylpiperidin-4-yl)phenyl)acrylamide (**9g**)

White solid, yield: 81%. ^1^H NMR (400 MHz, CDCl_3_) *δ*: 9.72 (s, 1H), 8.31 (d, *J* = 8.8 Hz, 1H), 8.08 (d, *J* = 10.1 Hz, 2H), 8.04 (s, 1H), 7.51 (s, 1H), 7.44 (d, *J* = 8.8 Hz, 1H), 7.32 (s, 1H), 6.85 (d, *J* = 16.6 Hz, 1H), 6.43 (dt, *J* = 28.3, 13.3 Hz, 2H), 5.76 (d, *J* = 9.6 Hz, 1H), 3.84 (s, 3H), 3.30 (d, *J* = 8.9 Hz, 2H), 3.15~3.00 (m, 2H), 2.77 (s, 1H), 2.56 (d, *J* = 19.9 Hz, 5H), 2.29~2.15 (m, 2H), 1.92 (dd, *J* = 25.6, 14.0 Hz, 2H), 1.23 (s, 3H). ^13^C NMR (100 MHz, CDCl_3_) *δ*: 165.0, 157.0, 155.4, 154.9, 147.1, 138.4, 133.6, 132.1, 130.8, 129.7, 128.5, 127.6, 126.5, 126.3, 124.9, 116.5, 108.0, 106.3, 56.0, 55.6, 47.6, 44.2, 34.8, 29.6, 7.4. [M + H]^+^ calculated for C_28_H_32_N_6_O_3_SCl_2_, 603.1706; found, 603.1701. Purity: 95.0% (by HPLC). ^1^H NMR, ^3^C NMR, HRMS, and HPLC of **9g** are shown in Figures S20, S21, S34 and S44, respectively.

##### *N*-(5-((5-Chloro-4-((2-(isopropylsulfinyl)phenyl)amino)pyrimidin-2-yl)amino)-4-methoxy-2-(1-methylpiperidin-4-yl)phenyl)acrylamide (**9h**)

White solid, yield: 56%. ^1^H NMR (400 MHz, CDCl_3_) *δ*: 9.96 (s, 1H), 8.42 (s, 1H), 8.34 (d, *J* = 8.3 Hz, 1H), 8.10 (s, 1H), 8.00 (s, 1H), 7.52~7.45 (m, 2H), 7.29 (d, *J* = 7.6 Hz, 1H), 7.06 (t, *J* = 7.5 Hz, 1H), 6.81 (s, 1H), 6.52~6.31 (m, 2H), 5.71 (d, *J* = 10.9 Hz, 1H), 3.83 (s, 3H), 3.38 (s, 2H), 3.26 (dt, *J* = 13.6, 6.8 Hz, 1H), 2.84~2.71 (m, 3H), 2.66 (s, 3H), 2.35 (d, *J* = 12.7 Hz, 2H), 1.96 (dd, *J* = 32.0, 13.1 Hz, 2H), 1.32 (d, *J* = 6.8 Hz, 3H), 1.09 (d, *J* = 6.8 Hz, 3H). ^13^C NMR (100 MHz, CDCl_3_) *δ*: 165.0, 157.0, 155.5, 154.6, 147.1, 140.2, 132.7, 132.2, 131.1, 127.9, 127.1, 126.6, 126.4, 123.7, 122.9, 116.5, 107.9, 106.4, 99.9, 56.1, 55.4, 52.8, 43.6, 34.2, 29.6, 16.3, 15.8. [M + H]^+^ calculated for C_29_H_35_N_6_O_3_SCl, 583.2253; found, 583.2255. Purity: 99.2% (by HPLC). ^1^H NMR, ^3^C NMR, HRMS, and HPLC of **9h** are shown in Figures S22, S23, S35 and S45, respectively.

##### *N*-(5-((5-Chloro-4-((4-fluoro-2-(isopropylsulfinyl)phenyl)amino)pyrimidin-2-yl)amino)-4-methoxy-2-(1-methylpiperidin-4-yl)phenyl)acrylamide (**9i**)

White solid, yield: 40%. ^1^H NMR (400 MHz, CDCl_3_) *δ*: 9.46 (s, 1H), 8.43 (s, 1H), 8.26~8.18 (m, 1H), 8.05 (s, 1H), 8.00 (s, 1H), 7.49 (s, 1H), 7.23 (dd, *J* = 11.4, 5.1 Hz, 1H), 7.12~7.04 (m, 1H), 6.80 (s, 1H), 6.49 (dd, *J* = 16.9, 10.2 Hz, 1H), 6.32 (d, *J* = 16.8 Hz, 1H), 5.70 (d, *J* = 10.2 Hz, 1H), 3.82 (s, 3H), 3.32 (s, 2H), 3.21 (dd, *J* = 13.6, 6.8 Hz, 1H), 3.00 (q, *J* = 7.3 Hz, 2H), 2.83 (t, *J* = 11.6 Hz, 1H), 2.61 (s, 3H), 2.25 (d, *J* = 12.0 Hz, 2H), 2.02~1.84 (m, 2H), 1.29 (d, *J* = 7.3 Hz, 3H), 1.13 (d, *J* = 6.8 Hz, 3H). ^13^C NMR (100 MHz, CDCl_3_) *δ*: 165.0, 159.1, 157.1, 156.7, 155.5, 154.7, 147.0, 135.6, 133.1, 130.9, 127.7, 127.2, 126.4, 119.0 (d, *J* = 22.0 Hz), 116.5, 114.4 (d, *J* = 24.2 Hz), 107.9, 105.9, 56.0, 55.4, 53.0, 45.8, 34.3, 16.4, 15.4, 8.5. [M + H]^+^ calculated for C_29_H_34_N_6_O_3_FSCl, 601.2158; found, 601.2166. Purity: 97.4% (by HPLC). ^1^H NMR, ^3^C NMR, HRMS, and HPLC of **9i** are shown in Figures S24, S25, S36 and S46, respectively.

##### *N*-(5-((5-Chloro-4-((4-chloro-2-(isopropylsulfinyl)phenyl)amino)pyrimidin-2-yl)amino)-4-methoxy-2-(1-methylpiperidin-4-yl)phenyl)acrylamide (**9j**)

White solid, yield: 60%. ^1^H NMR (400 MHz, CDCl_3_) *δ*: 9.95 (s, 1H), 8.42~8.31 (m, 2H), 8.09 (s, 1H), 8.02 (s, 1H), 7.52~7.42 (m, 2H), 7.27 (d, *J* = 7.3 Hz, 1H), 6.81 (s, 1H), 6.48 (dd, *J* = 16.8, 10.1 Hz, 1H), 6.36 (d, *J* = 16.7 Hz, 1H), 5.74 (d, *J* = 10.0 Hz, 1H), 3.83 (s, 3H), 3.30 (dd, *J* = 12.8, 6.8 Hz, 3H), 2.79 (d, *J* = 11.3 Hz, 1H), 2.60 (s, 5H), 2.22 (d, *J* = 12.1 Hz, 2H), 1.92 (t, *J* = 15.4 Hz, 2H), 1.35 (d, *J* = 6.8 Hz, 3H), 1.14 (d, *J* = 6.7 Hz, 3H). ^13^C NMR (100 MHz, CDCl_3_) *δ*: 165.1, 156.9, 155.2, 154.8, 147.0, 138.9, 133.5, 132.0, 130.8, 127.9, 127.7, 127.6, 127.5, 127.4, 126.3, 124.6, 116.6, 107.9, 106.3, 56.0, 55.5, 53.1, 44.1, 34.6, 30.2, 16.4, 15.7. [M + H]^+^ calculated for C_29_H_34_N_6_O_3_SCl_2_, 617.1863; found, 617.1878. Purity: 97.7% (by HPLC). ^1^H NMR, ^3^C NMR, HRMS, and HPLC of **9j** are shown in Figures S26, S27, S37 and S47, respectively.

### 3.2. Cell Lines and Culture

The human cancer cell lines (H1975, H2228) used in this study were purchased from the Guangzhou ginny ou biotechnology Co., Ltd. (Guangzhou, China). The human cancer cell lines (NCI-H522) used in this study were purchased from the Shenzhen Haodi Huatuo Biotechnology Co., LTD (Shenzhen, China). The human cancer cell lines (LOVO, HLF, BJ, LO2) used in this study were purchased from the Laboratory Animal Service Center at Sun Yat-sen University (Guangzhou, China). Cell lines were cultivated in 1640 containing 10% (*v/v*) heat-inactivated fetal bovine serum (FBS), 100 units/mL penicillin, and 100 μg/mL streptomycin. Cell lines LOVO, HLF, BJ, and LO2 were cultivated in DMEM containing 10% (*v/v*) heat-inactivated fetal bovine serum (FBS), 100 units/mL penicillin, and 100 μg/mL streptomycin.

### 3.3. CCK8 Assays

A total of 7000 cells per well were seeded in a 96-well plate with at least three technical replicates; then, exposed to different concentrations (10, 5, 1, 0.1, 0.05, 0.01 uM) of the test compounds for 72 h. After the treatment, CCK8 (Sigma, St. Louis, MO, USA) was added to each well under sterile conditions (to a final concentration of 10% of the total volume) and the plates were incubated for 2–4 h at 37 °C. Absorbance was measured at 450 nm in a microplate reader. The cytotoxic effects of each compound were expressed as IC_50_ values, which represent the drug concentrations required to cause 50% tumour cell growth inhibition, and calculated with GraphPad Prism Software version 5.02 (GraphPad Inc., La Jolla, CA, USA).

### 3.4. Cell Apoptosis

To assay the number of apoptotic cells, cells were stained with allo-phycocyanin–annexin V (#550475, BD Pharmingen, Becton Drive Franklin Lakes, NJ, USA) for 15 min; then, they were washed and resuspended in 1:500 propidium iodide (#81845, Sigma-Aldrich, St. Louis, Mo, USA) viability marker, and acquired on LSRFortessa. Data were analyzed with FlowJo software. Annexin V+ cells were considered as apoptotic cells.

### 3.5. Wound-Healing Assay

H1975 cells were plated in a six-well culture dish at 5 × 10^4^ cells/dish and grown for 24 h, and thenonmigrated cells were scraped off the upper surface of the membrane with a 200 μL pipet. The medium was then replaced with a serum-free 1640 medium and treated with compound **9j** at the indicated concentrations for another 24 h or 48 h. After the samples were washed with phosphate buffer solution (PBS), the cell images were immediately detected by a Zeiss LSM 570 laser scanning confocal microscope (Carl Zeiss, Jena, Germany).

### 3.6. In Vitro Cell Migration and Invasion Assay

Migration assay was performed using an 8 um pore Boyden chamber (24-well, #353097, Corning, NY, USA), and incubated in the presence or absence of compound **9j**. A total of 50,000 cells were seeded in serum-free medium in the top chamber and allowed to migrate for 48 h toward the bottom chamber containing 10% fetal bovine serum medium. Cells were fixed and permeabilized with cold 70% ethanol and methanol, and stained with 0.4% crystal violet. The average number of migrating cells was evaluated from at least four independent microscope fields.

### 3.7. Molecular Docking

All the docking studies were performed by Schrödinger suites (Maestro v.9.0, Schrödinger Inc., New York, NY, USA). For ligand preparation, 3D structures of all the ligands were constructed using ChemBio3D ultra14.0 and then, the initial lowest energy conformations were calculated with ligPrep. For protein preparation, the X-ray crystal structures of kinases were downloaded from the RCSB PDB Bank (available online: http://www.rcsb.org/ (accessed on 2 February 2023)); then, Protein Preparation Wizard was used with default settings to pre-process and refine. For dockings, the grid center was placed at the centroid of the ligand-binding site; and then, Glide with SP protocol was used to perform the docking study. The docking poses were affirmed by PyMOL (www.pymol.org, accessed on 2 February 2023) and MOE 2018.01 [36].

### 3.8. Western Blot Analysis

H1975 (H2228) cells seeded in 60 mm dishes at a density of 5 × 10^5^ cells/well were incubated with or without compound **9j** (0, 5, 10, 100 nM) for 2–4 h. After treatment, cells were stained as previously described [37].

## 4. Conclusions

In the process of cancer activation, it depends not only on a single signal pathway, but also on multiple interacting signal pathways. Single-target drugs regulate the main signal pathways of tumor; however, due to the complex relationship between signal pathways, it may also promote tumor cells to reactivate through other signal pathways, resulting in drug resistance. In addition, more and more cancer patients with two or more carcinogenic gene mutations are discovered in recent years. Based on the above, developing multi-target drugs is still one of the effective strategies for treating cancer.

ALK and EGFR are extremely effective targets in the process of tumor therapy. Targeting ALK and EGFR can effectively improve the quality of life and survival time of cancer patients. The currently used ALK inhibitors and EGFR inhibitors have produced drug-resistant mutations, affecting the clinical use of existing inhibitors. Dual-target inhibitors affect two different pathways of disease progression, often producing synergistic or enhancing effects and reducing the development of disease resistance.

To explore the application of multi-target drugs in anti-tumor, we synthesized ten new dual-target inhibitors targeting EGFR and ALK simultaneously with ceritinib as the lead molecular. The optimal compound **9j** exhibited excellent activity for both EGFR ^T790M/L858R^, EML4-ALK kinases, and inhibition activity against tumor cell proliferation. The other assay results for compound **9j**, such as low toxicity to normal cells and effective inhibition of tumor cell migration and invasion, indicate that **9j** is worthy of further study.

## Figures and Tables

**Table 1:** Anti-proliferative activities of compounds **9a**–**9j**.

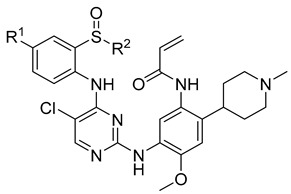				
IC_50_ (μM) ^a^	R^1^, R^2^	H1975	H2228	H522
**9a**	R^1^ = H, R^2^ = cyclopropyl	0.4511 ± 0.07	0.1214 ± 0.09	>10
**9b**	R^1^ = H, R^2^ = methyl cyclopropyl	0.06895 ± 0.007	0.09895 ± 0.007	>10
**9c**	R^1^ = F, R^2^ = methyl cyclopropyl	0.2707 ± 0.13	0.1027 ± 0.07	>10
**9d**	R^1^ =Cl, R^2^ =methyl cyclopropyl	0.1143 ± 0.08	0.089 ± 0.03	>10
**9e**	R^1^ = H, R^2^ = ethyl	0.1699 ± 0.02	0.0977 ± 0.05	>10
**9f**	R^1^ = F, R^2^ = ethyl	0.5495 ± 0.09	0.0928 ± 0.06	>10
**9g**	R^1^ = Cl, R^2^ = ethyl	0.06901 ± 0.01	0.1037 ± 0.05	>10
**9h**	R^1^ = H, R^2^ = *iso*-propyl	0.08389 ± 0.03	0.0766 ± 0.02	>10
**9i**	R^1^ = F, R^2^ = *iso*-propyl	0.3094 ± 0.11	0.09808 ± 0.06	>10
**9j**	R^1^ = Cl, R^2^ = *iso*-propyl	0.07829 ± 0.03	0.08183 ± 0.02	>10
**Ceritinib**		0.07829 ± 0.03	0.0221 ± 0.008	/
**Osimertinib**		0.0041 ± 0.001	2.016 ± 0.008	/

^a^ The anti-proliferative activities of the compounds were assessed by the CCK8 assay. Data are averages of at least three independent determinants.

**Table 2:** Toxicity to normal cells ^a^.

Compound.	9j	Selectivity Ratio ^b^	Osimertinib	Selectivity Ratio ^b^
LO2	23.96 ± 0.03	306.1	1.72 ± 0.05	419.5
HK2	25.71 ± 0.08	329.6	2.13 ± 0.09	519.5
HLF	27.87 ± 0.01	357.3	3.24 ± 0.08	790.2
293A	20.003 ± 0.05	255.5	2.25 ± 0.02	548.8
HUVEC	21.36 ± 0.06	273.8	4.34 ± 0.01	1058.5
H1975	0.07829 ± 0.03	/	0.0041 ± 0.001	/

^a^ Data are presented as the mean ± SE from the dose-response cures of at least three independent experiments. ^b^ Selectivity ratio (IC_50_ human normal cells)/(IC_50_ H1975).

## Data Availability

The data are available upon request to the authors.

## Supplementary Materials

The following supporting information can be downloaded at: https://www.mdpi.com/article/10.3390/molecules28052006/s1. Figure S1: HE staining of mouse lungs and hearts, Figures S2–S27: NMR spectra of compounds, Figures S28–S47: HRMS spectra of compounds.

## Sample Availability

Samples of the compounds **9a**–**9j** are available from the authors.
