# Supplementary material for: Novel Dual-Target Kinase Inhibitors of EGFR and ALK Were Designed, Synthesized, and Induced Cell Apoptosis in Non-Small Cell Lung Cancer
Source: Molecules. 2023 Feb 21;28(5):2006. doi: 10.3390/molecules28052006 (PMC10004195; doi:10.3390/molecules28052006)
Supplement: Supplementary file 1 [file molecules-28-02006-s001.zip › molecules-2201780-supplementary.pdf]

Novel Dual-Target Kinase Inhibitors of EGFR and ALK Were Designed, Synthesized, and Induced Cell Apoptosis in Non-Small Cell Lung Cancer

Wei Li [2,†], Yangyang Fan [1,†], Wenyan Nie [1], Han Yao [2], Yuanyuan Ren [2], Mengxuan Wang [1], Haoran Nie [1], Chenxi Gu [2], Jiadai Liu [2] and Baijiao An [1,3,*]

[1] School of Pharmacy, Binzhou Medical University, Yantai 264003, China

[2] School of Pharmaceutical Sciences, Sun Yat-Sen University, Guangzhou 510006, China

[3] Shandong Technology Innovation Center of Molecular Targeting and Intelligent Diagnosis and Treatment, Yantai 264003, China

Supplementary Contents

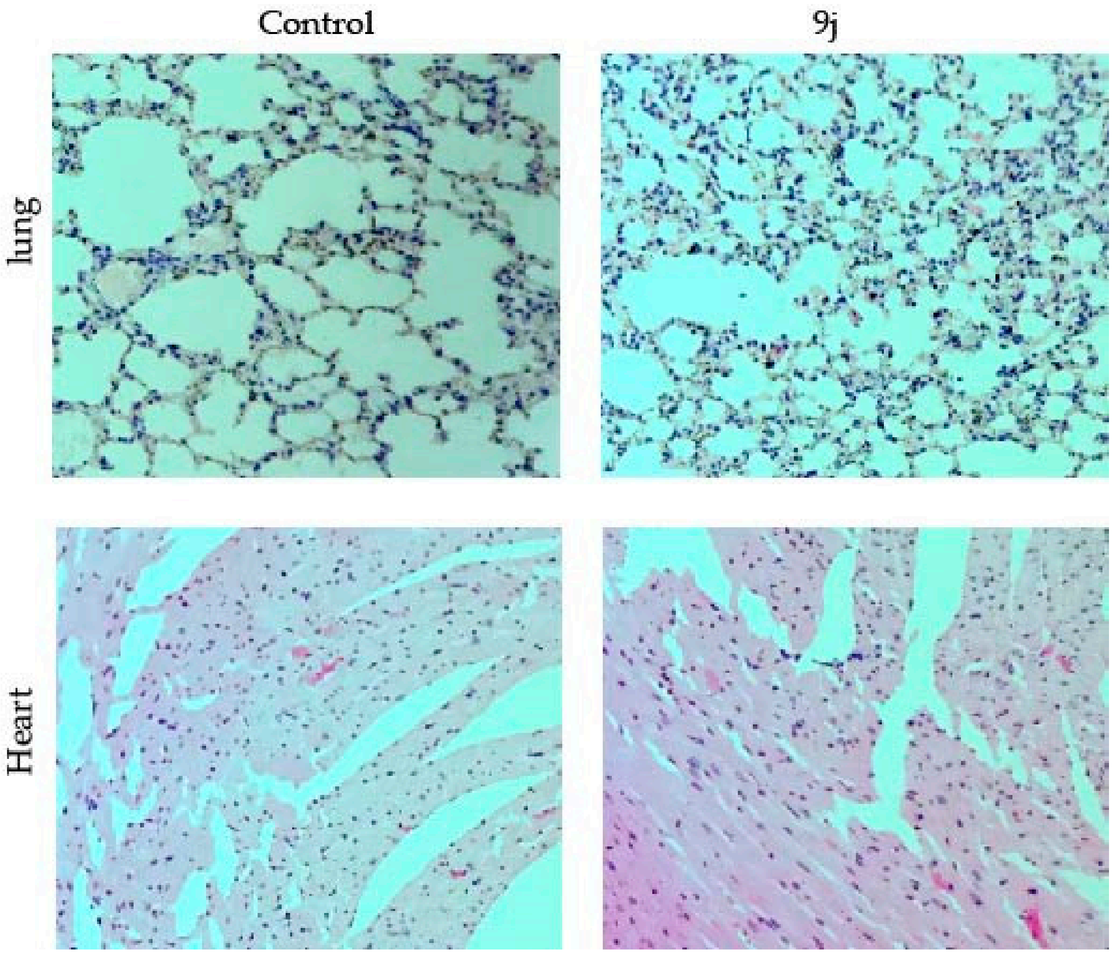

Figure S1. HE staining of mouse lungs and hearts

NMR spectra of compounds

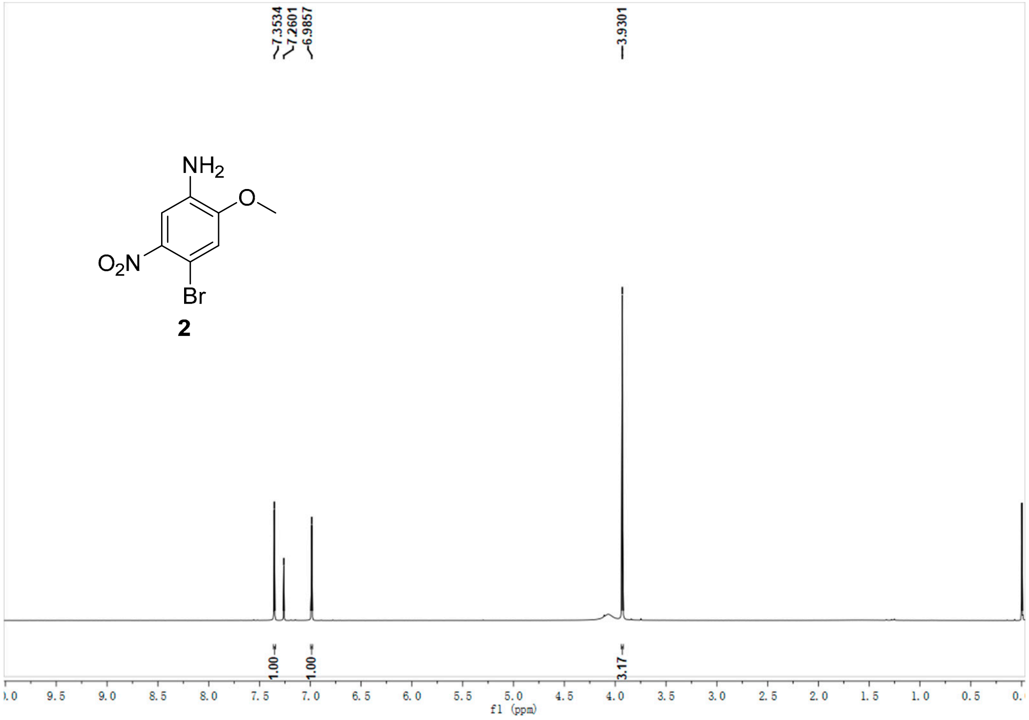

Figure S2. ^{1}H NMR Spectrum of **2** in $CDCl_3$

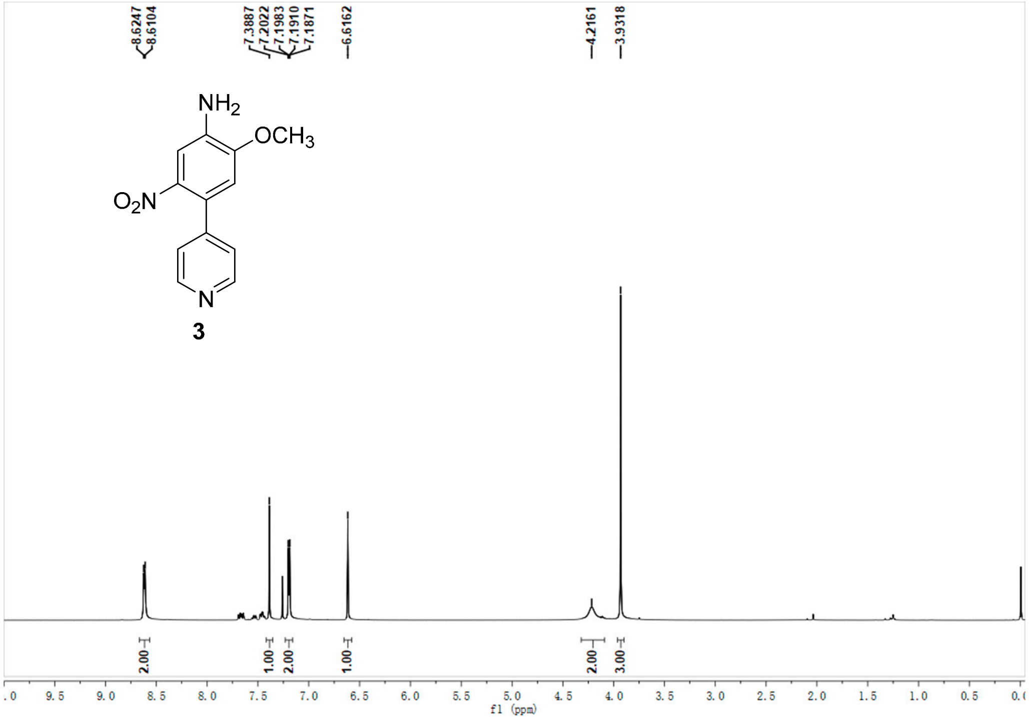

Figure S3. ^{1}H NMR Spectrum of **3** in $CDCl_3$

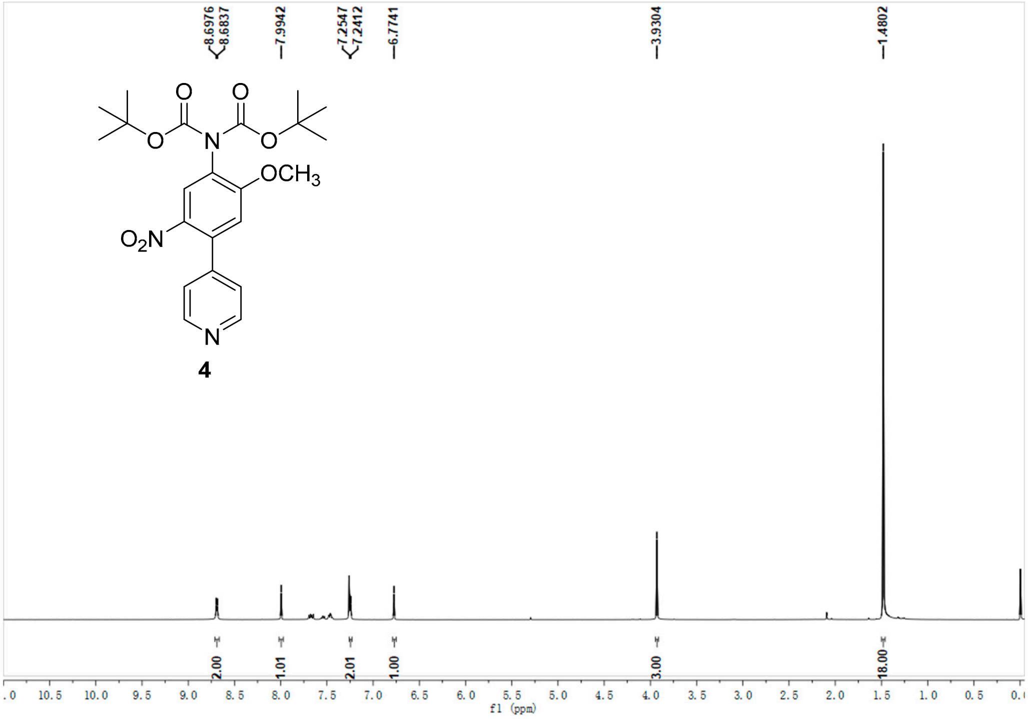

Figure S4. ^{1}H NMR Spectrum of **4** in $CDCl_3$

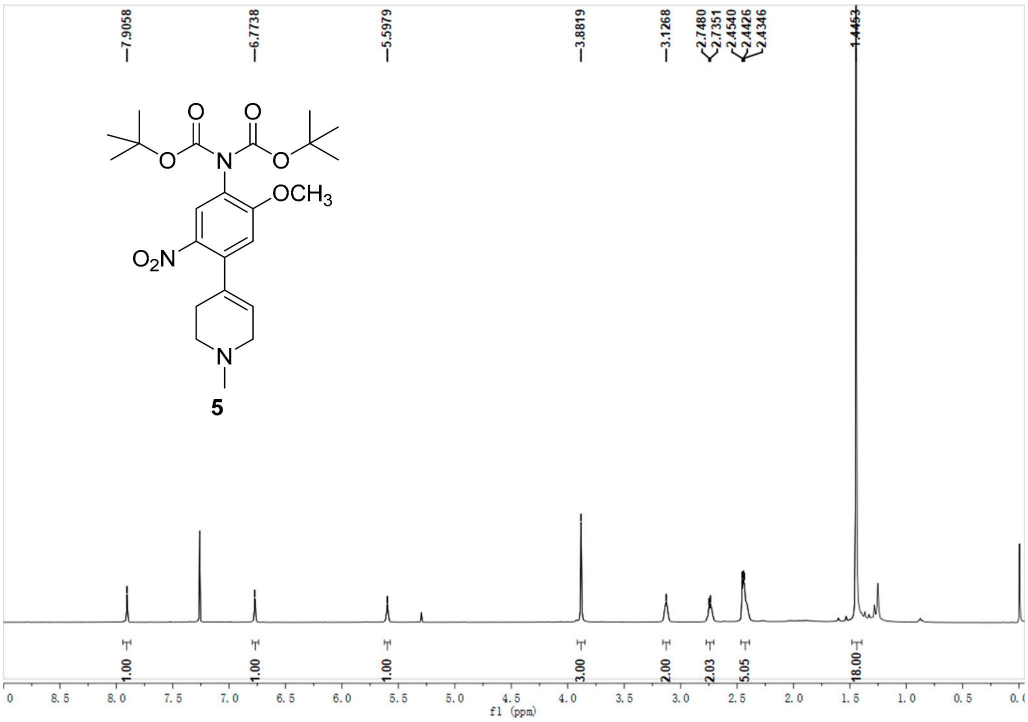

Figure S5. ^{1}H NMR Spectrum of **5** in $CDCl_3$

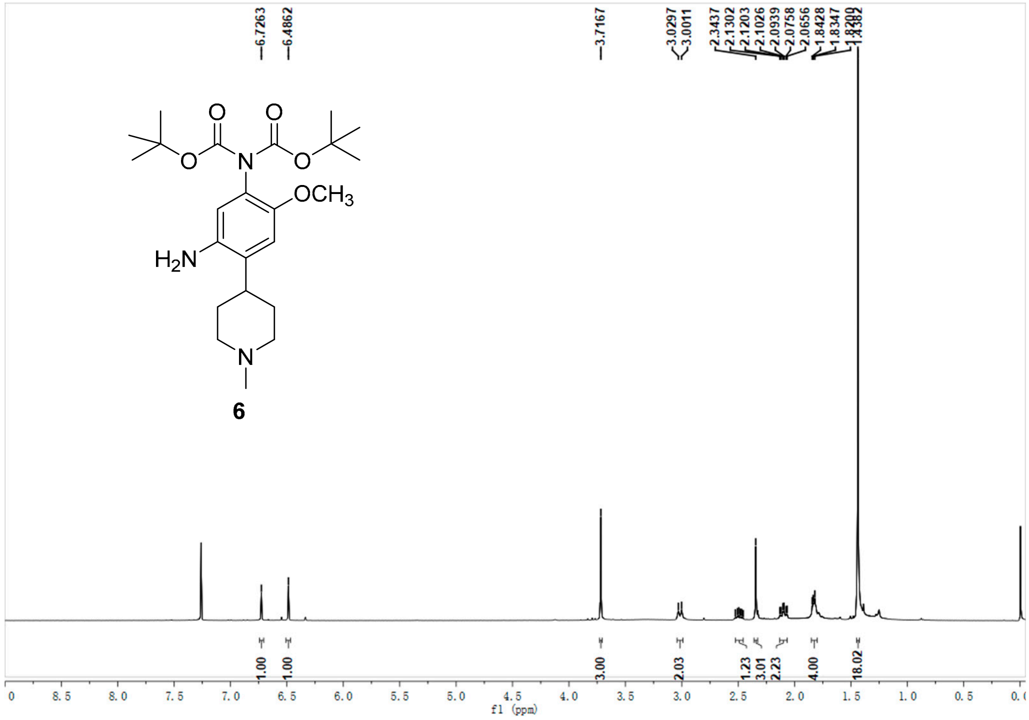

Figure S6. ¹H NMR Spectrum of **6** in CDCl₃

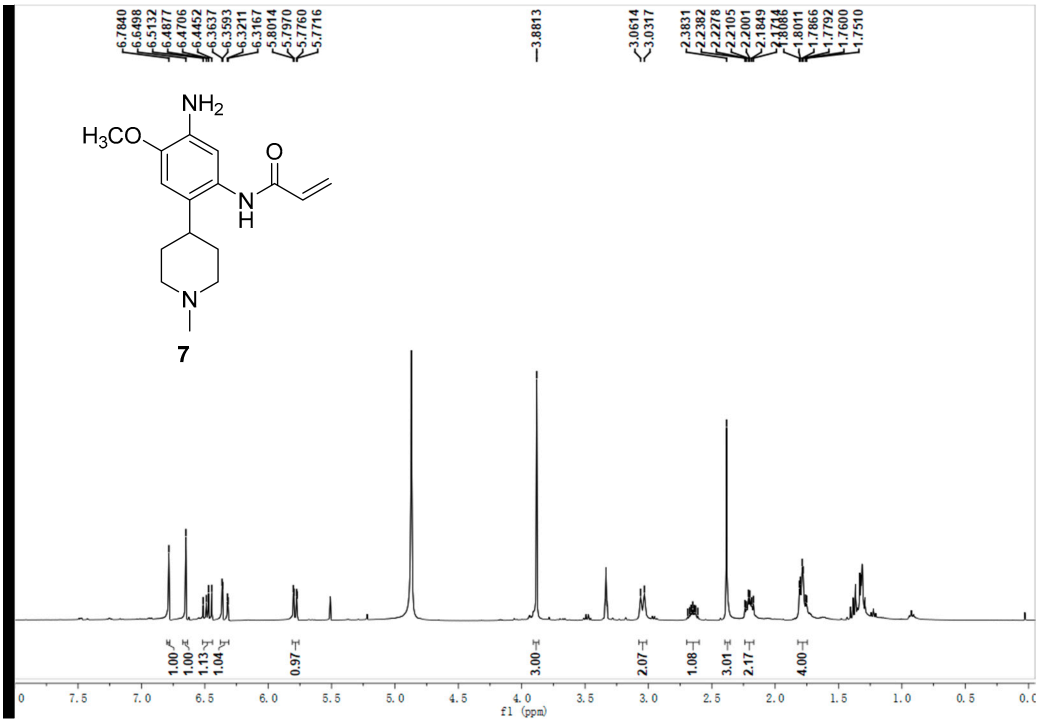

Figure S7. ¹H NMR Spectrum of **7** in CD₃OD

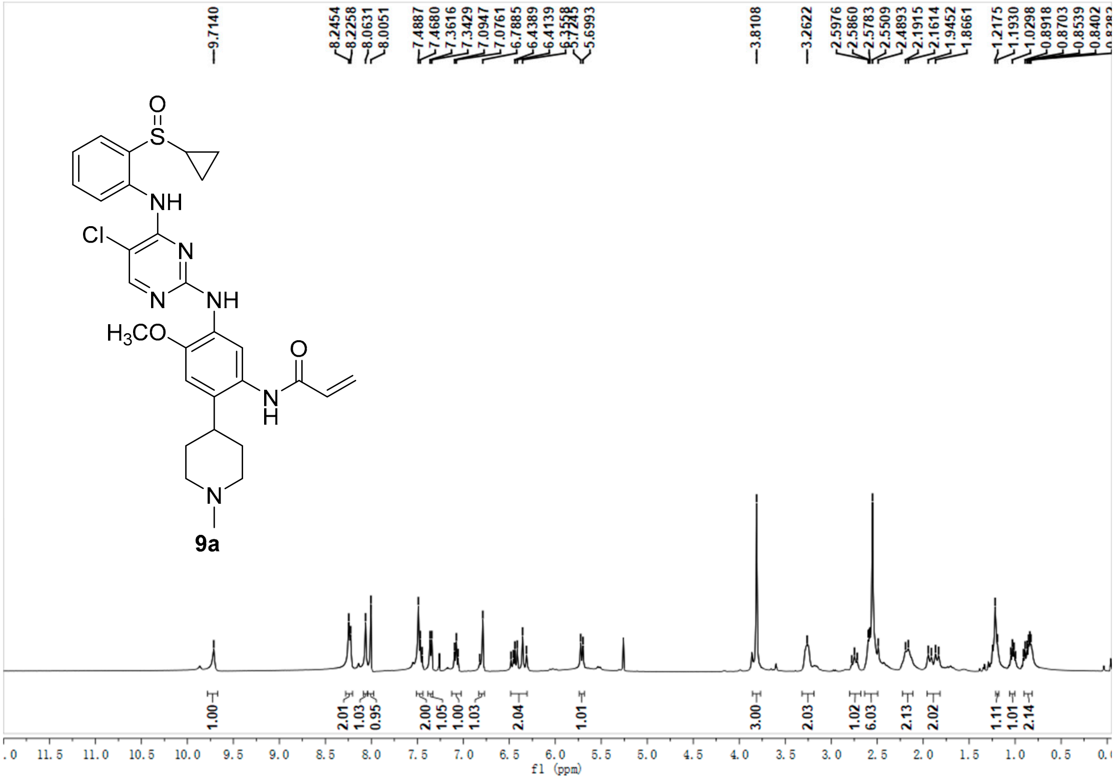

Figure S8. ¹H NMR Spectrum of **9a** in $CDCl_3$

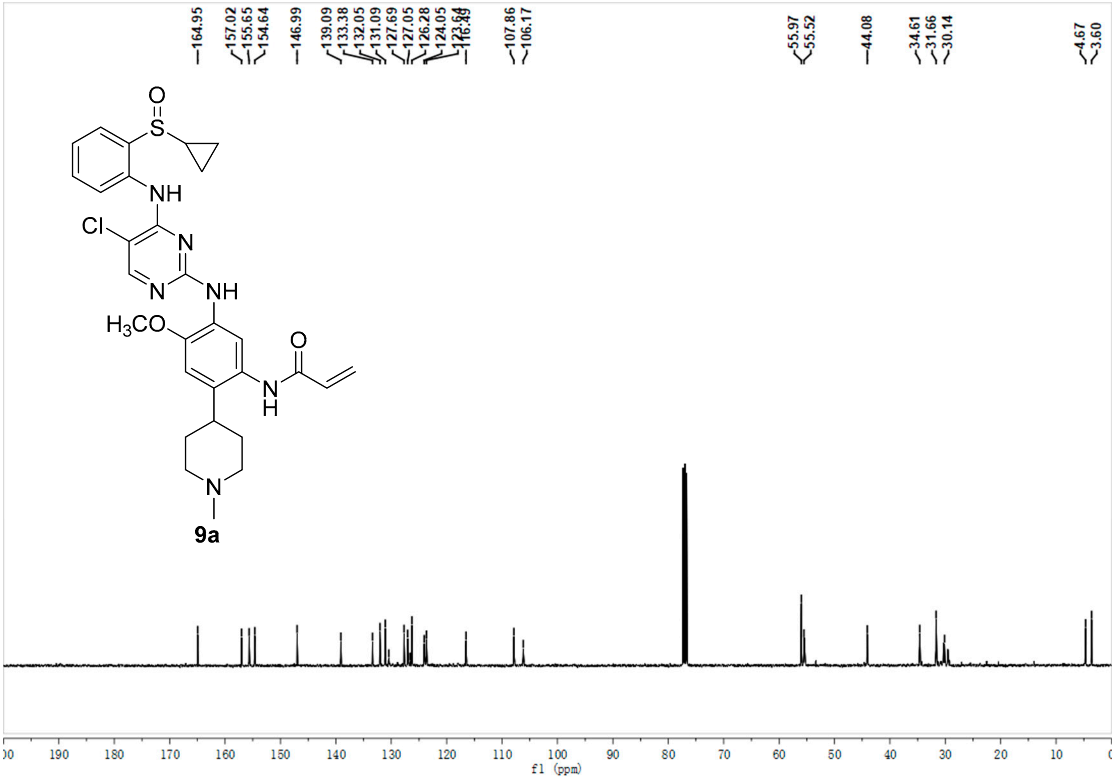

Figure S9. ¹³C NMR Spectrum of **9a** in $CDCl_3$

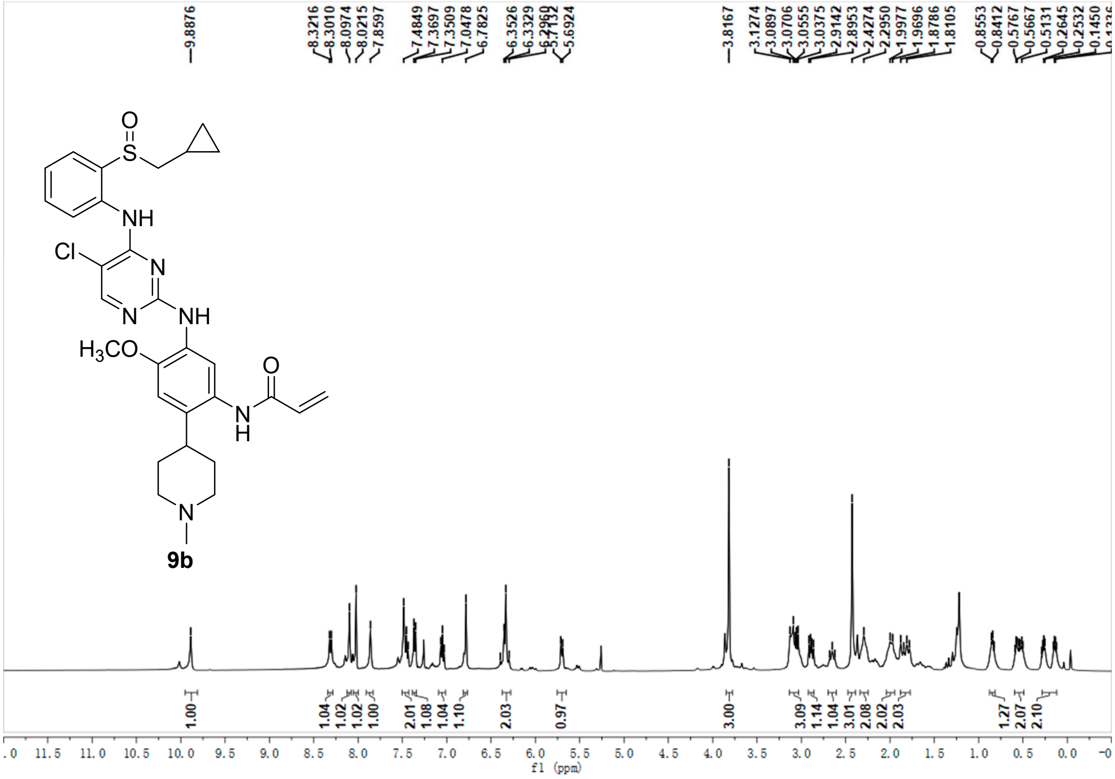

Figure S10. ^{1}H NMR Spectrum of **9b** in $CDCl_3$

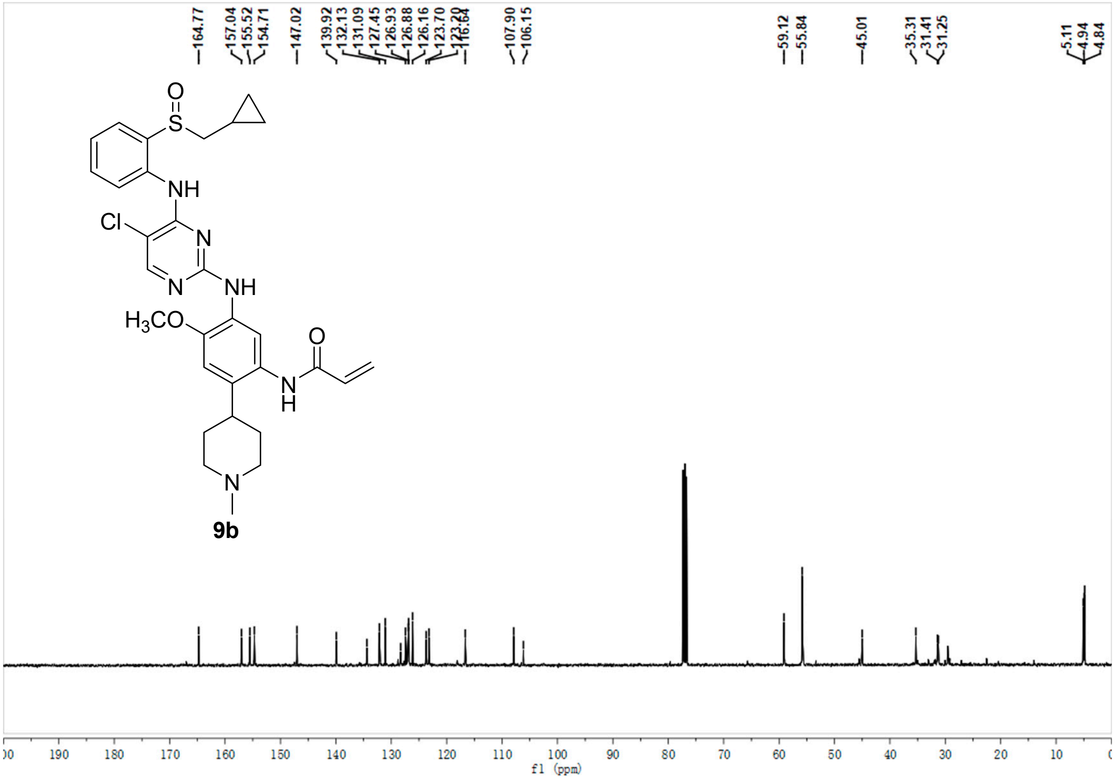

Figure S11. ^{13}C NMR Spectrum of **9b** in $CDCl_3$

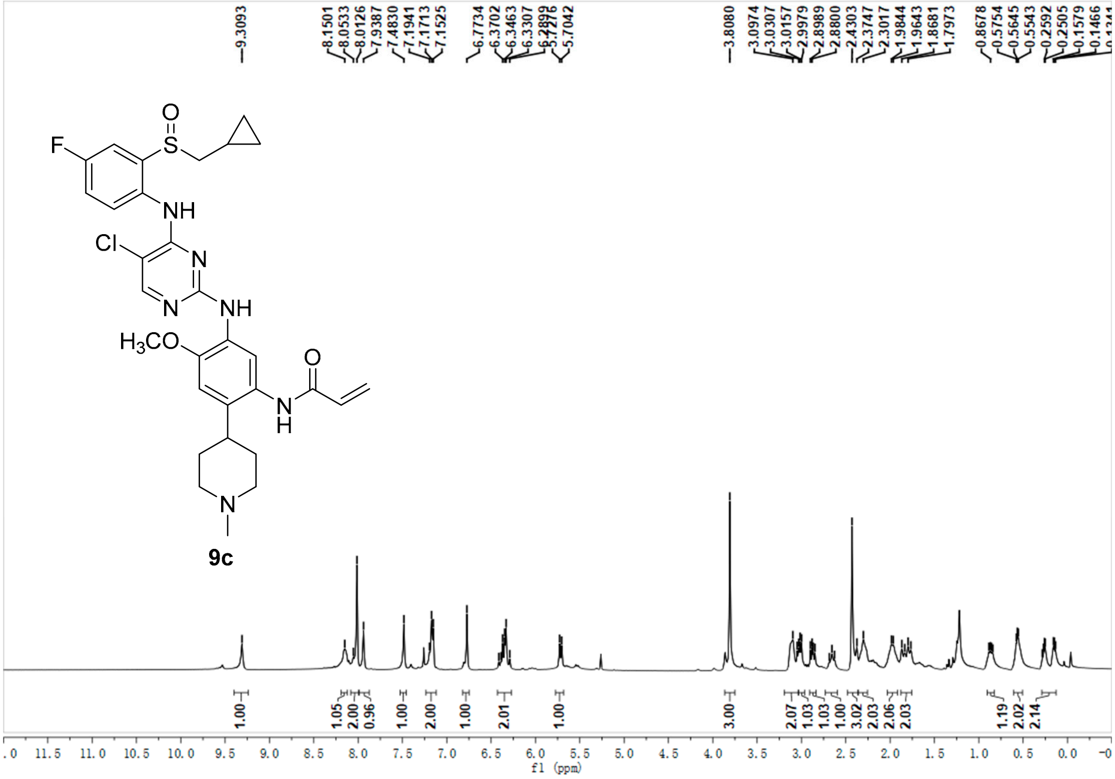

Figure S12. ^{1}H NMR Spectrum of **9c** in $CDCl_3$

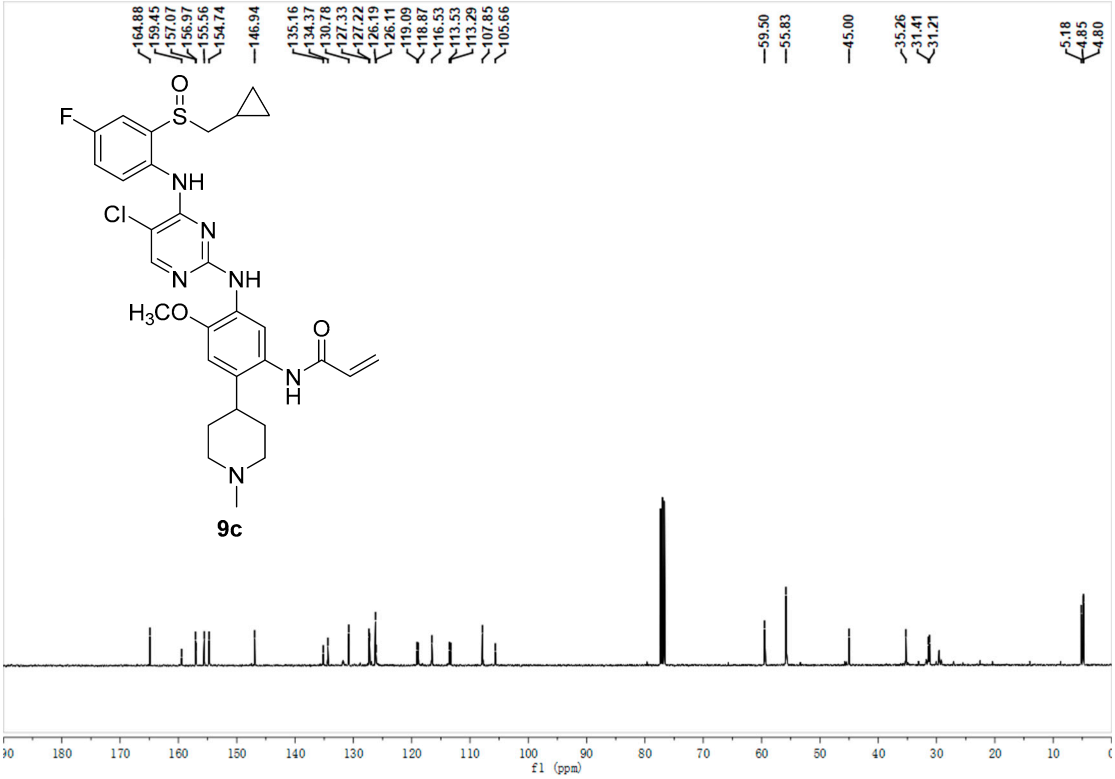

Figure S13. ^{13}C NMR Spectrum of **9c** in $CDCl_3$

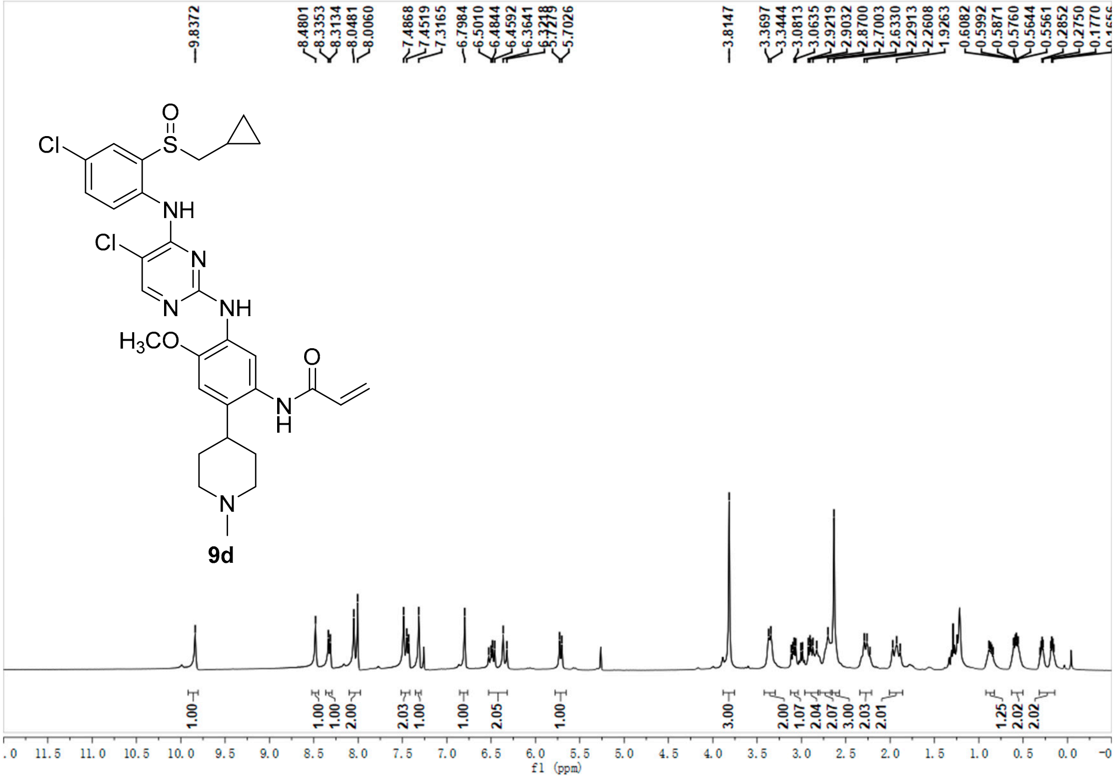

Figure S14. ^{1}H NMR Spectrum of **9d** in $CDCl_3$

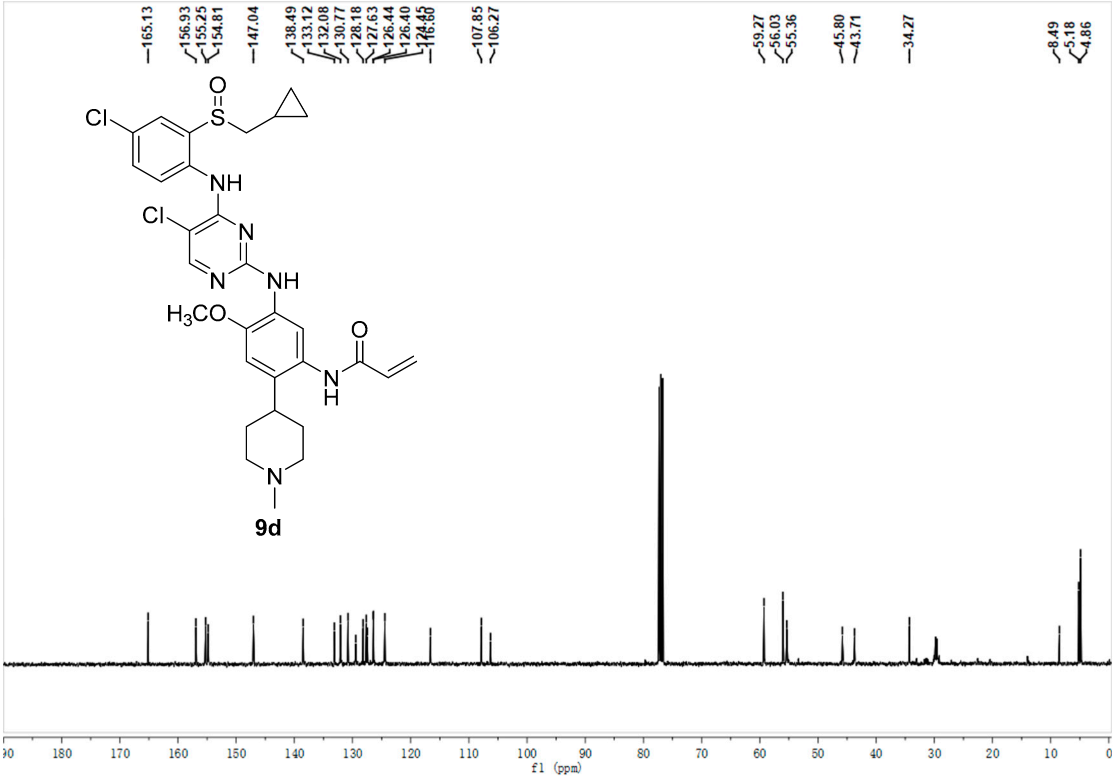

Figure S15. ^{13}C NMR Spectrum of **9d** in $CDCl_3$

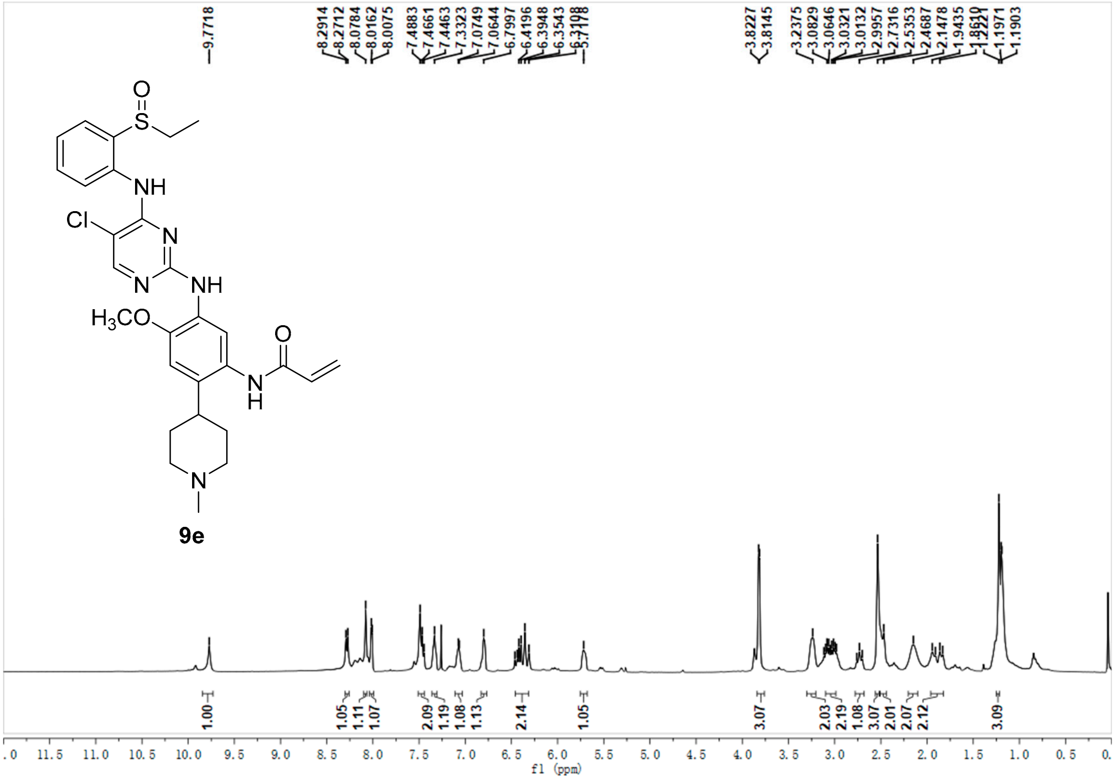

Figure S16. ^{1}H NMR Spectrum of **9e** in $CDCl_3$

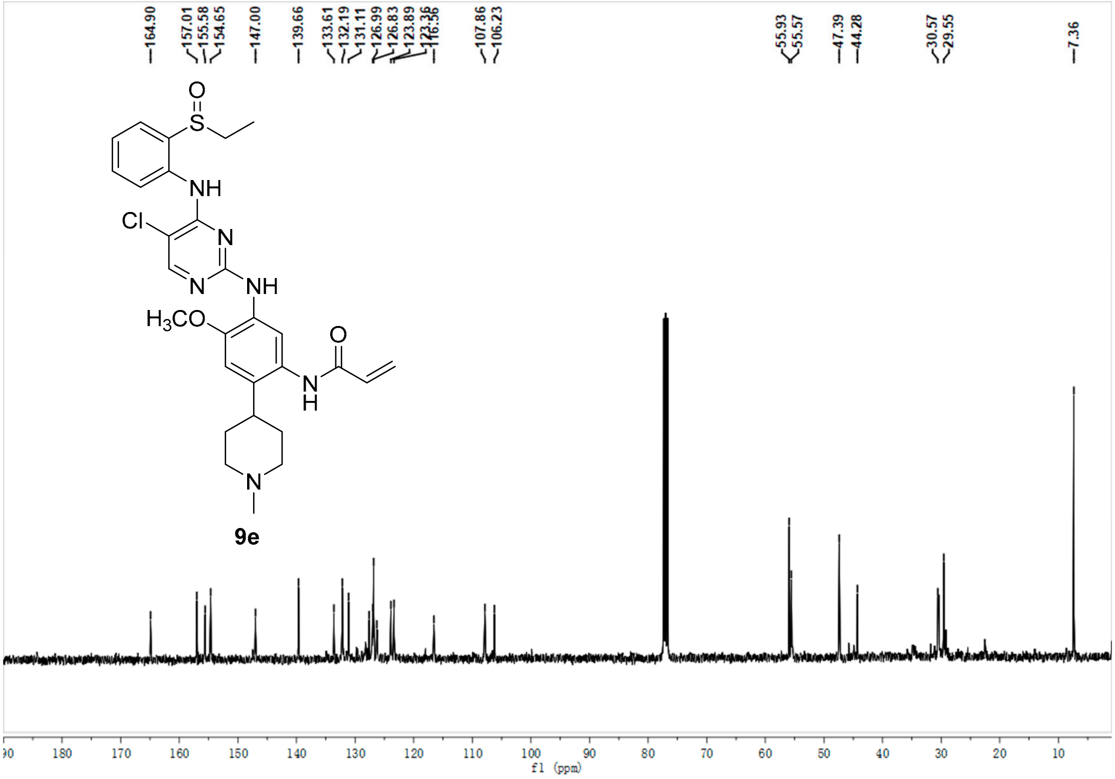

Figure S17. ^{13}C NMR Spectrum of **9e** in $CDCl_3$

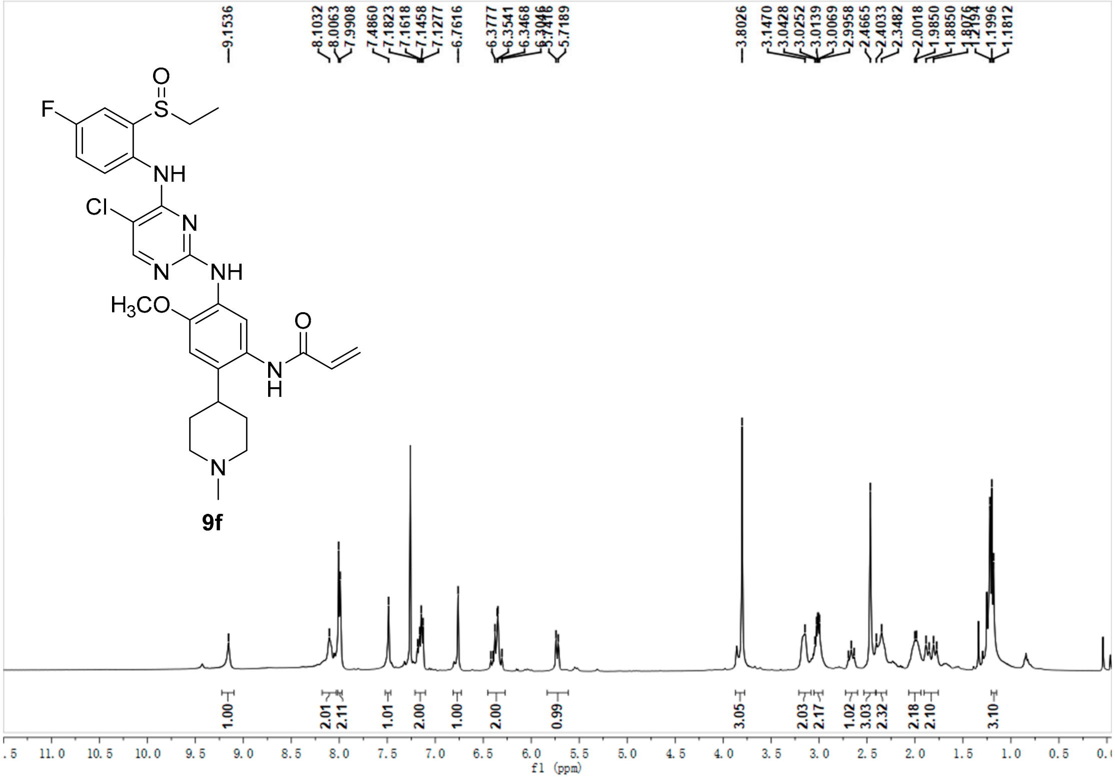

Figure S18. ^{1}H NMR Spectrum of **9f** in $CDCl_3$

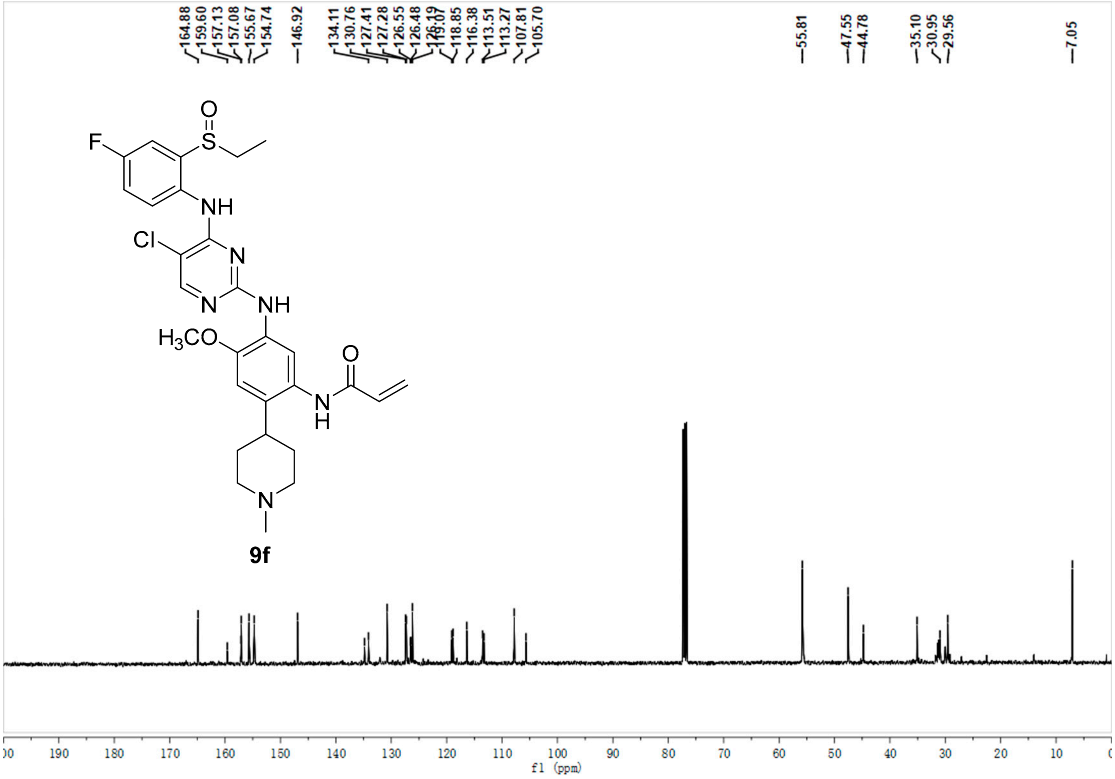

Figure S19. ^{13}C NMR Spectrum of **9f** in $CDCl_3$

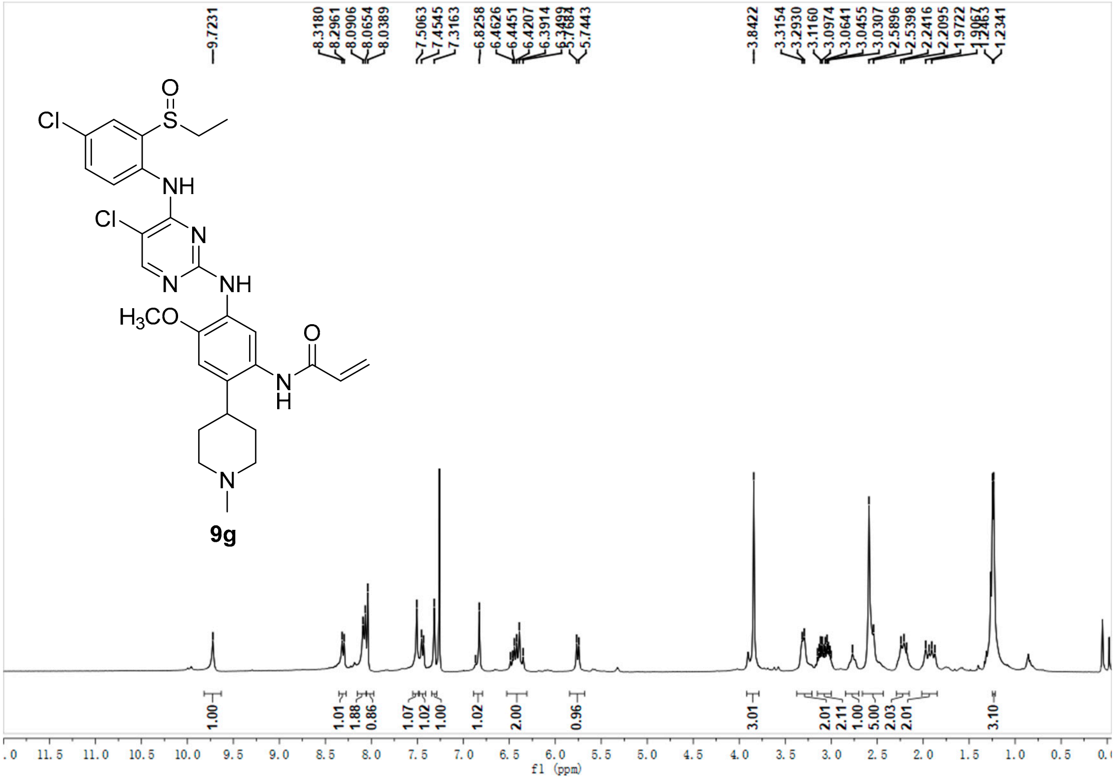

Figure S20. ^{1}H NMR Spectrum of **9g** in $CDCl_3$

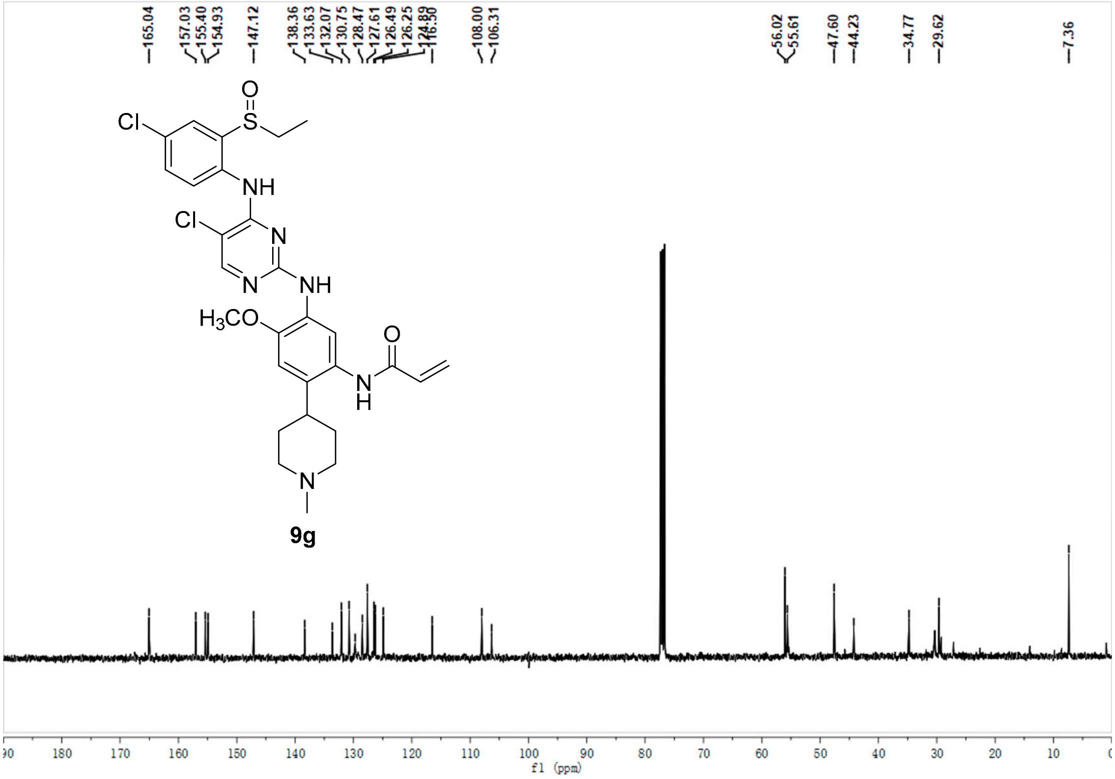

Figure S21. ^{13}C NMR Spectrum of **9g** in $CDCl_3$

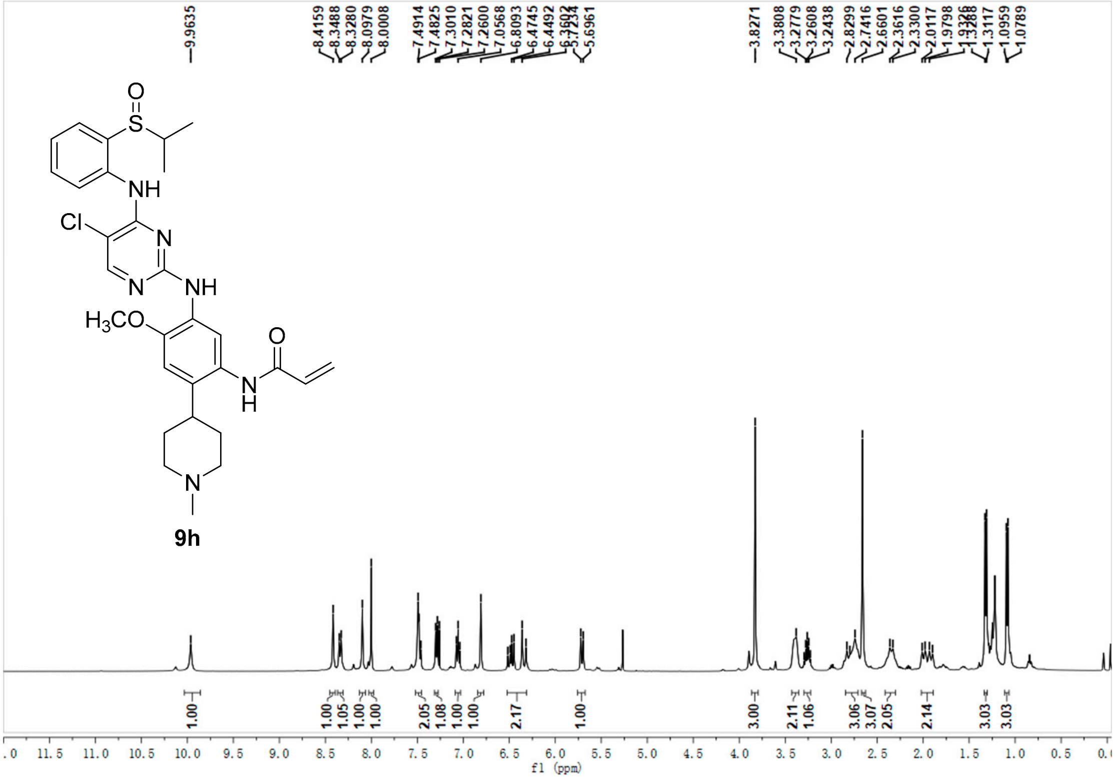

Figure S22. ^{1}H NMR Spectrum of **9h** in $CDCl_3$

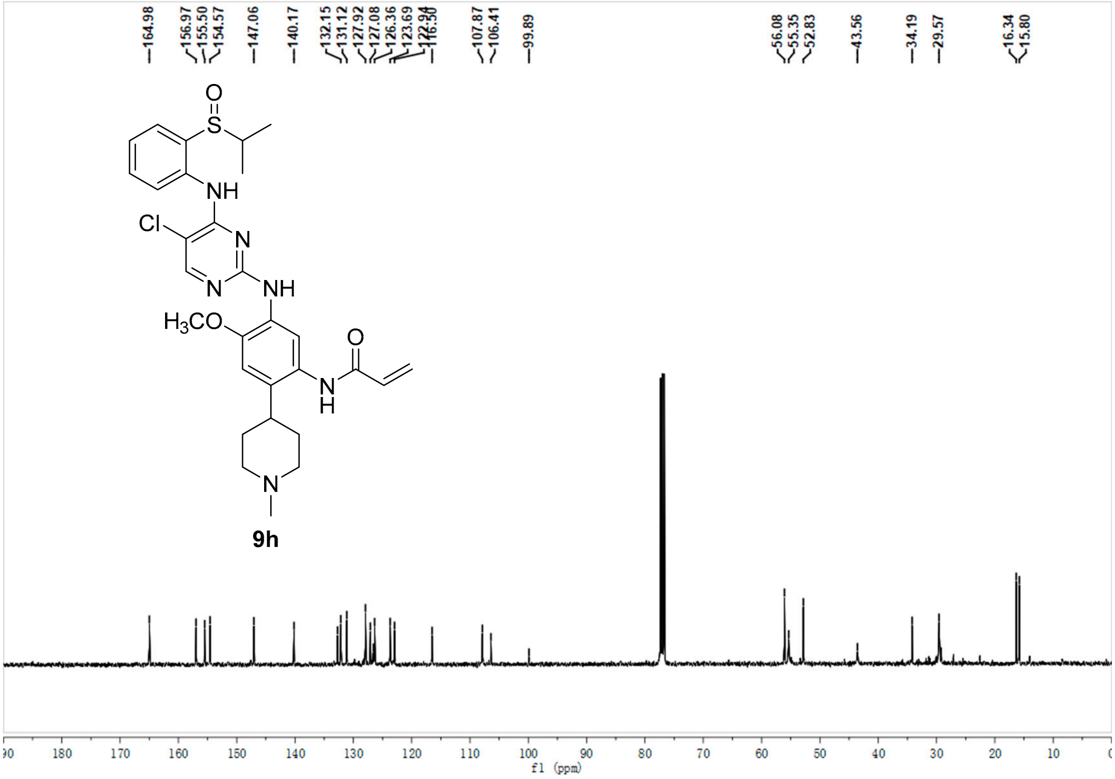

Figure S23. ^{13}CNMR Spectrum of **9h** in $CDCl_3$

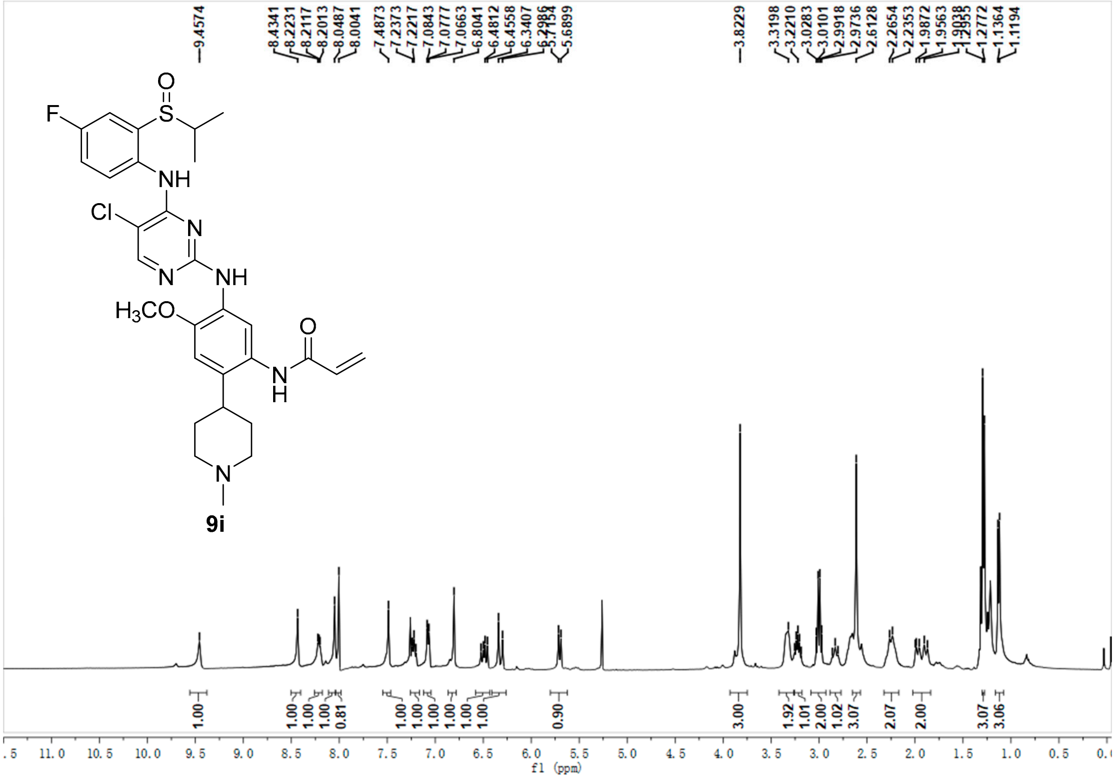

Figure S24. ^{1}H NMR Spectrum of **9i** in $CDCl_3$

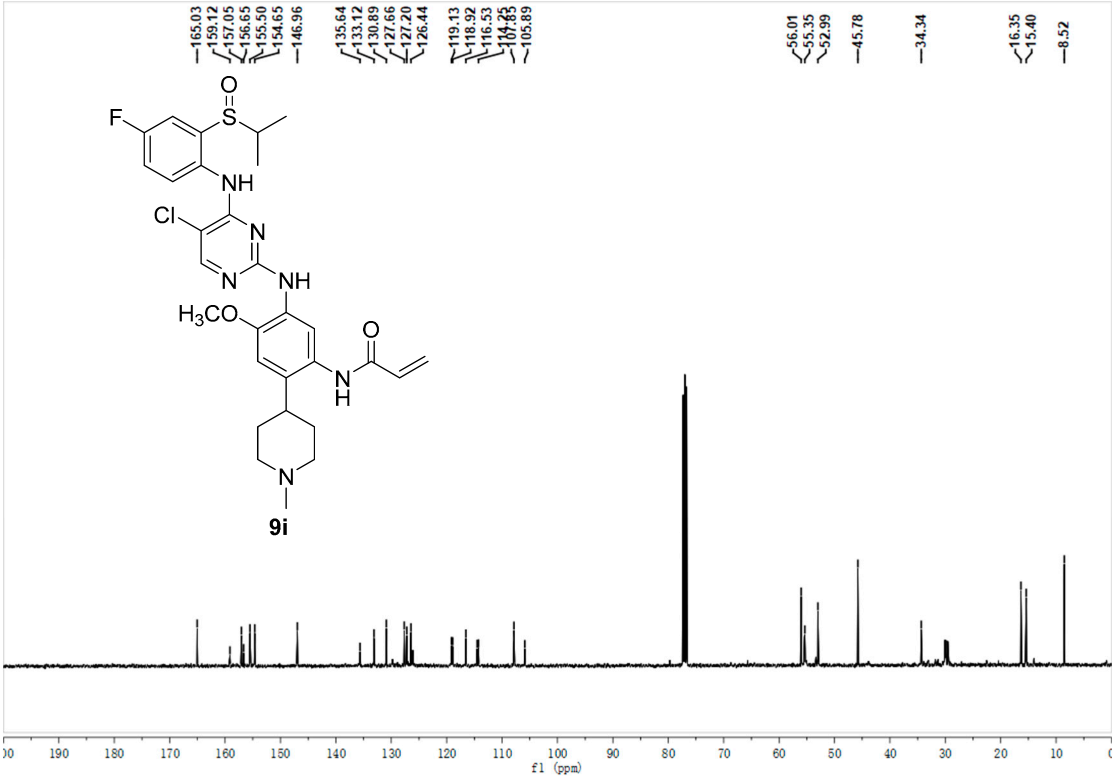

Figure S25. ^{13}C NMR Spectrum of **9i** in $CDCl_3$

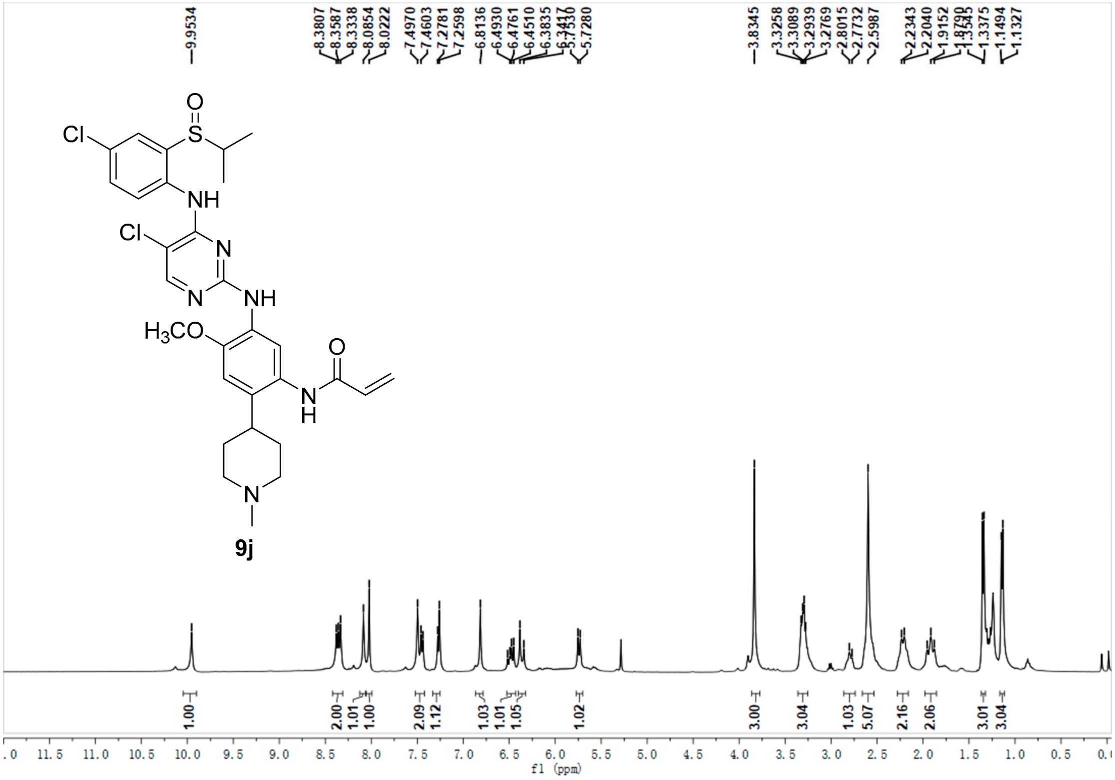

Figure S26. ^{1}H NMR Spectrum of **9j** in CDCl3

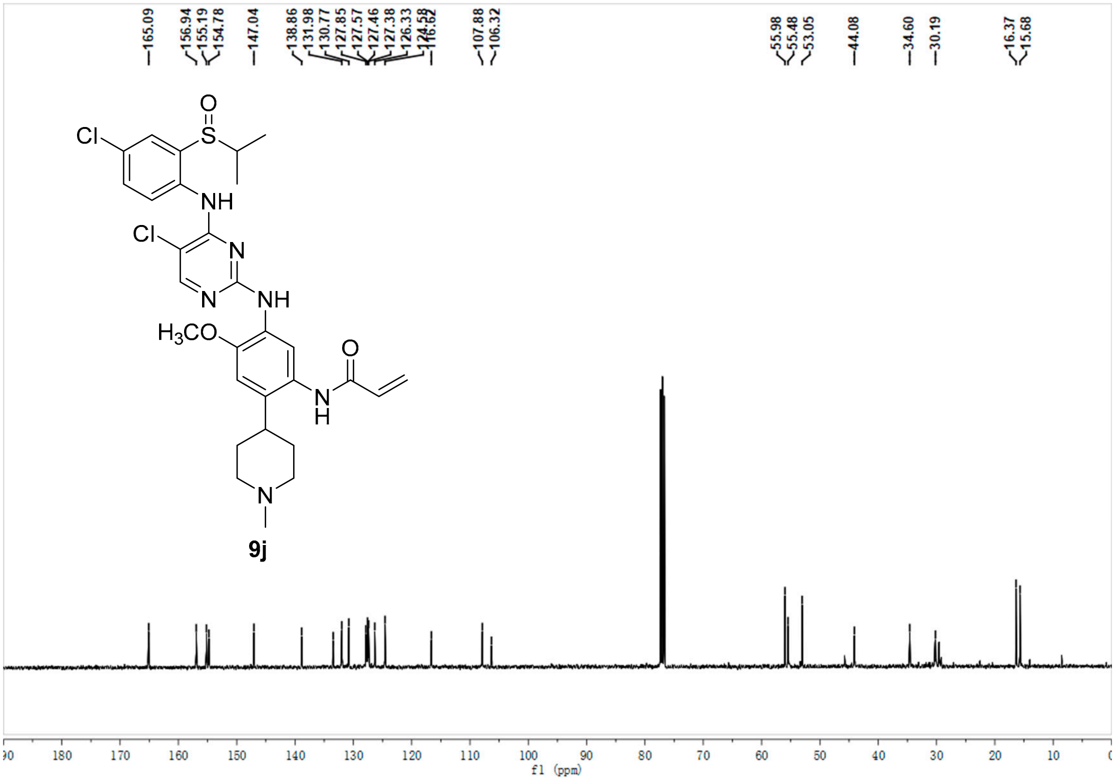

Figure S27. ^{13}C NMR Spectrum of **9j** in CDCl3

HRMS spectra of compounds

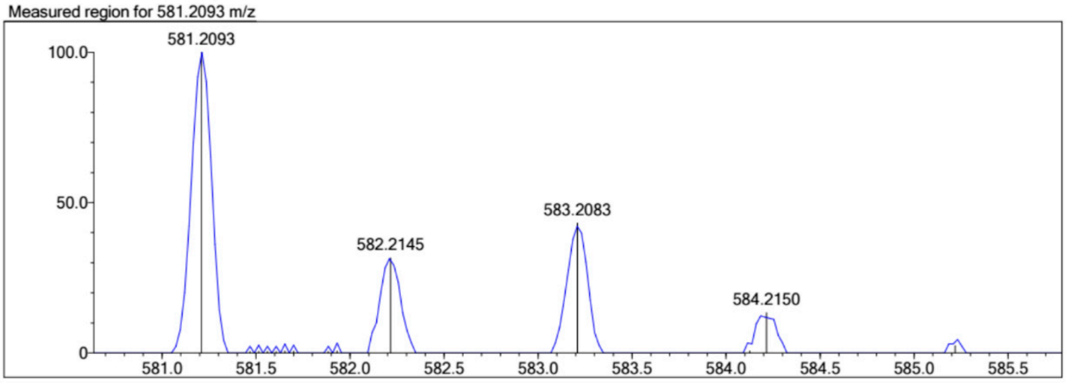

Figure S28. HRMS spectrum of **9a**

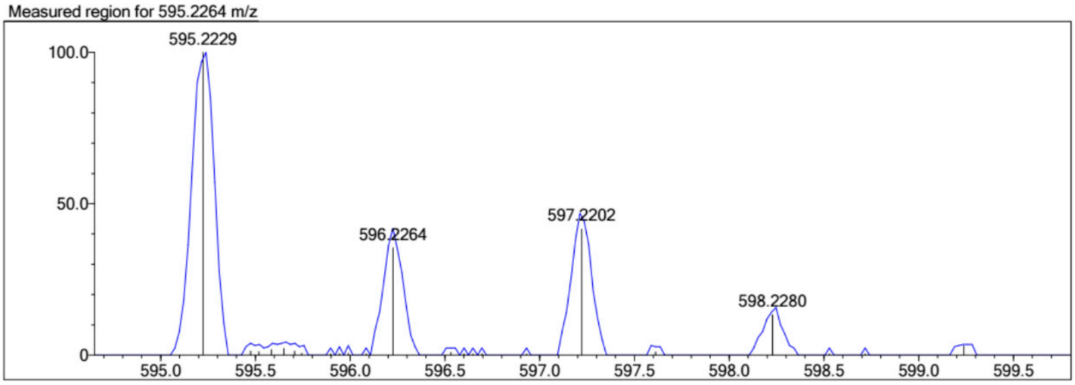

Figure S29. HRMS spectrum of **9b**

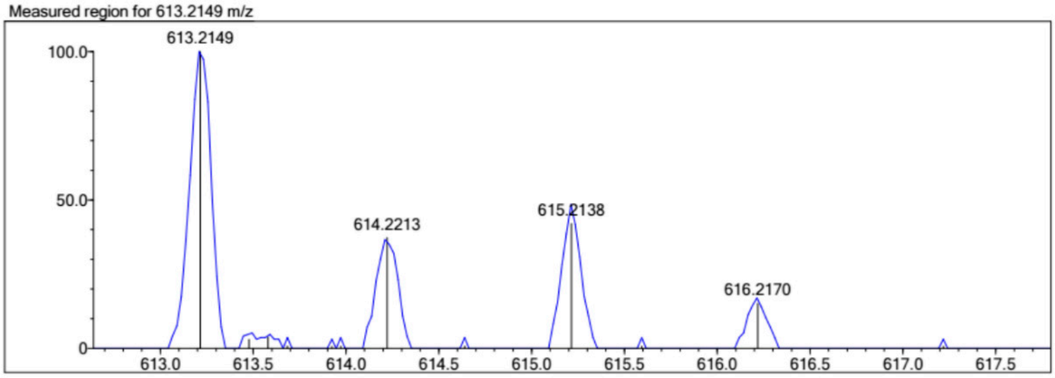

Figure S30. HRMS spectrum of **9c**

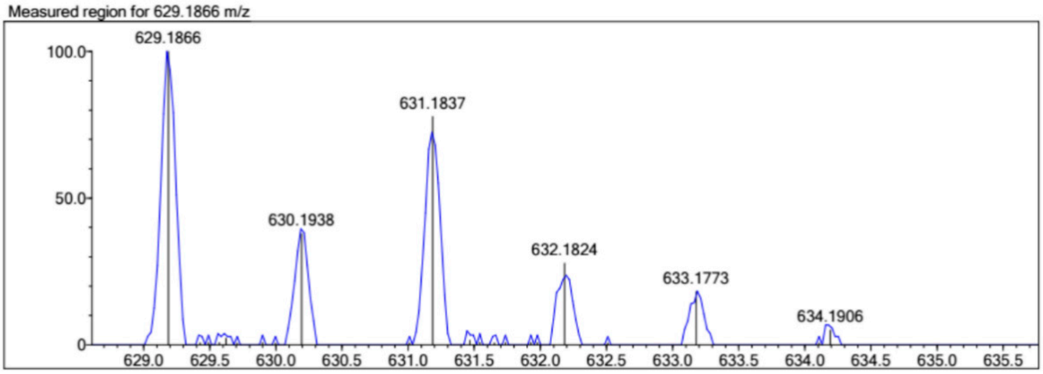

Figure S31. HRMS spectrum of **9d**

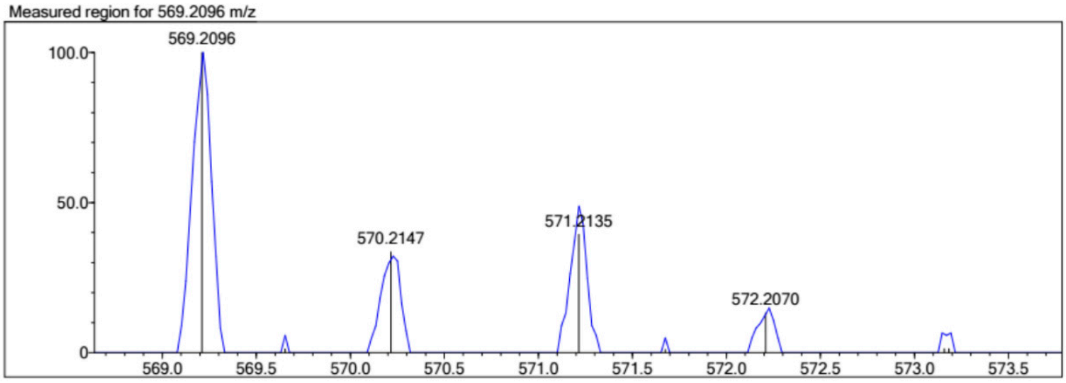

Figure S32. HRMS spectrum of **9e**

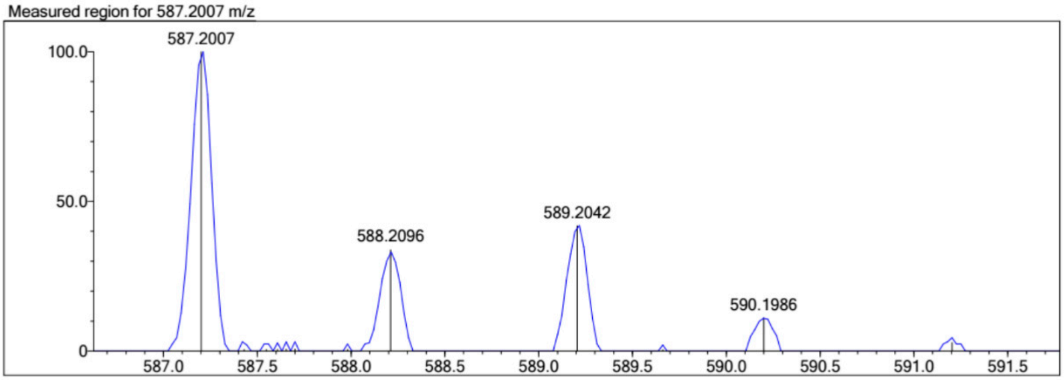

Figure S33. HRMS spectrum of **9f**

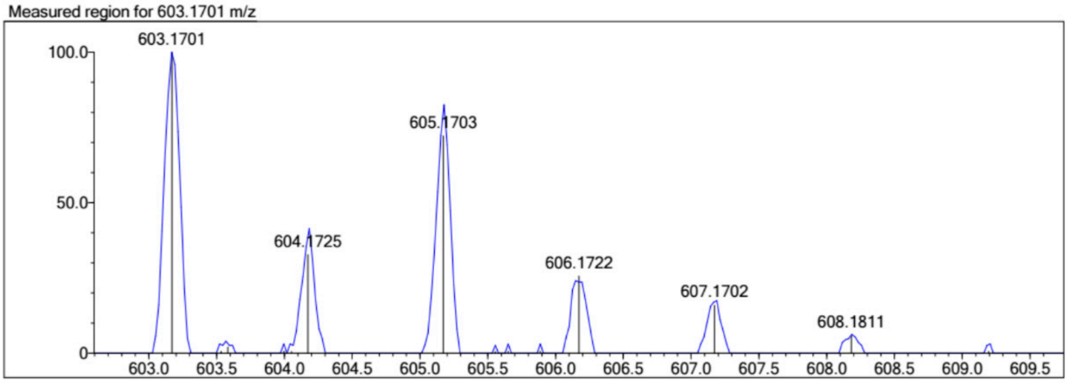

Figure S34. HRMS spectrum of **9g**

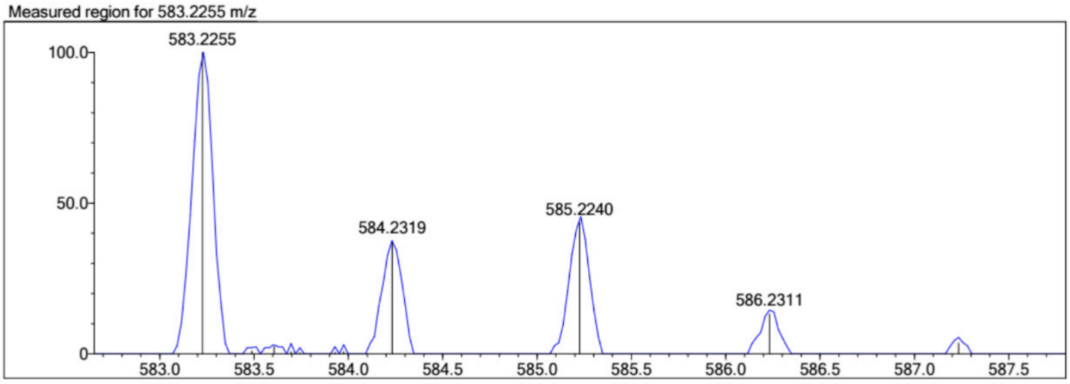

Figure S35. HRMS spectrum of **9h**

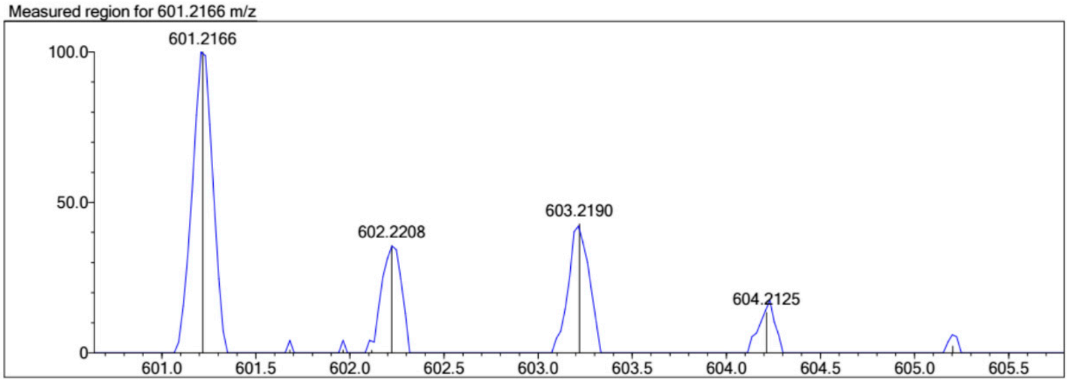

Figure S36. HRMS spectrum of **9i**

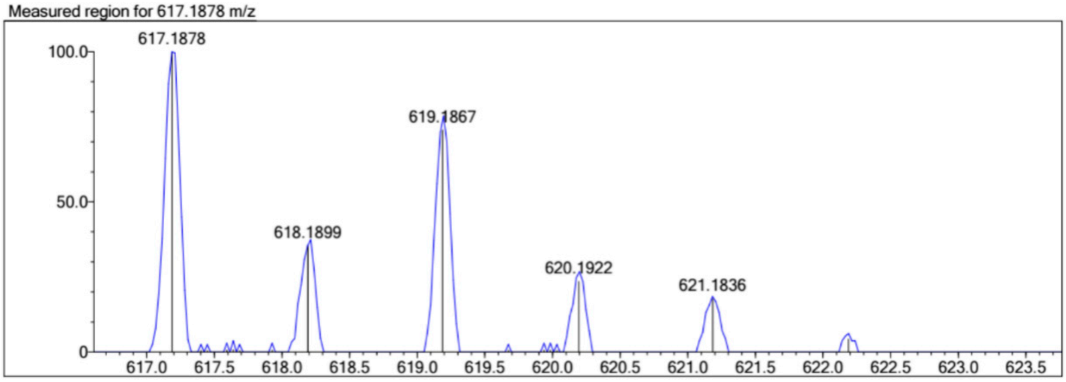

Figure S37. HRMS spectrum of **9j**

HPLC of compounds

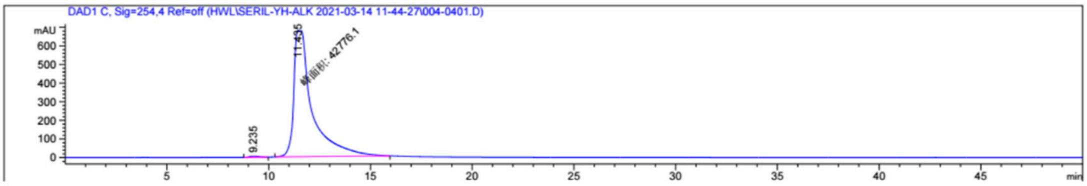

面积百分比报告

排序 : 信号
乘积因子: : 1.0000
稀释因子: : 1.0000
内标使用乘积因子和稀释因子

信号 1: DAD1 C, Sig=254,4 Ref=off

峰 #	保留时间 [min]	类型	峰宽 [min]	峰面积 [mAU*s]	峰高 [mAU]	峰面积 %
1	9.235	BB	0.4614	169.40498	5.59309	0.3945
2	11.435	MM T	1.0509	4.27761e4	678.39203	99.6055

总量 : 4.29455e4 683.98512

Figure S38. HPLC of **9a**

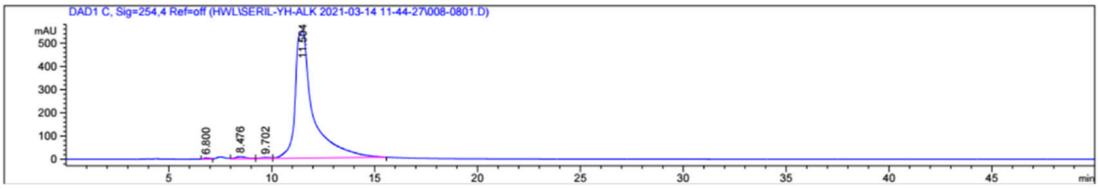

面积百分比报告

排序 : 信号
乘积因子: : 1.0000
稀释因子: : 1.0000
内标使用乘积因子和稀释因子

信号 1: DAD1 C, Sig=254,4 Ref=off

峰 #	保留时间 [min]	类型	峰宽 [min]	峰面积 [mAU*s]	峰高 [mAU]	峰面积 %
1	6.800	BV	0.2345	93.25786	5.48124	0.2748
2	8.476	VV	0.5365	325.57455	8.86421	0.9593
3	9.702	VV	0.4664	146.84416	4.25786	0.4327
4	11.504	VB	0.7805	3.33734e4	548.03192	98.3333

总量 : 3.39391e4 566.63523

Figure S39. HPLC of **9b**

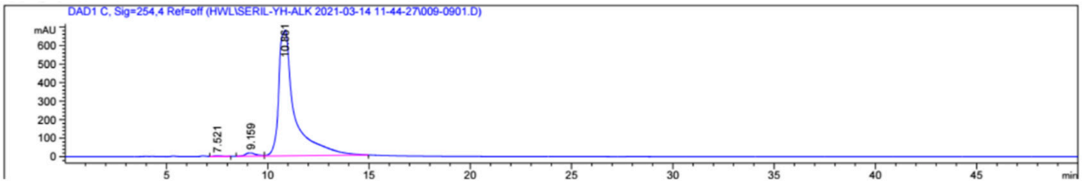

面积百分比报告

排序 : 信号
乘积因子: : 1.0000
稀释因子: : 1.0000
内标使用乘积因子和稀释因子

信号 1: DAD1 C, Sig=254,4 Ref=off

峰 #	保留时间 [min]	类型	峰宽 [min]	峰面积 [mAU*s]	峰高 [mAU]	峰面积 %
1	7.521	VB	0.3301	68.28077	2.80384	0.1808
2	9.159	BV	0.4888	623.66418	16.88924	1.6515
3	10.861	VB	0.7061	3.70719e4	677.82788	98.1677
总量 :				3.77639e4	697.52096	

Figure S40. HPLC of **9c**

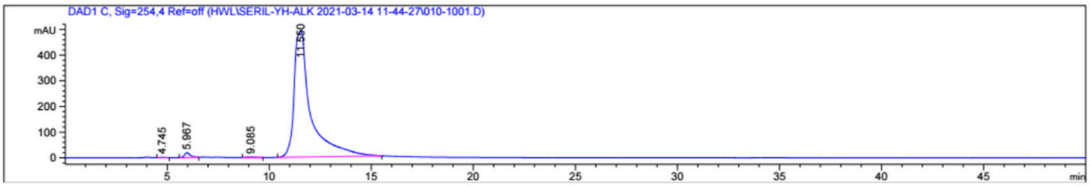

面积百分比报告

排序 : 信号
乘积因子: : 1.0000
稀释因子: : 1.0000
内标使用乘积因子和稀释因子

信号 1: DAD1 C, Sig=254,4 Ref=off

峰 #	保留时间 [min]	类型	峰宽 [min]	峰面积 [mAU*s]	峰高 [mAU]	峰面积 %
1	4.745	BB	0.2366	20.57413	1.20837	0.0725
2	5.967	BB	0.2914	360.23660	18.32386	1.2694
3	9.085	BB	0.3606	55.48083	1.88908	0.1955
4	11.550	BB	0.7370	2.79433e4	493.19931	98.4627
总量 :				2.83795e4	514.62062	

Figure S41. HPLC of **9d**

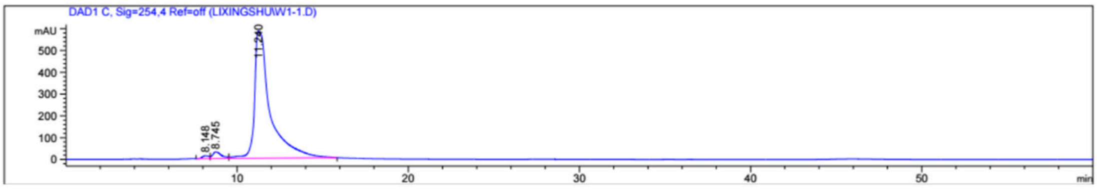

面积百分比报告

排序 : 信号
乘积因子: : 1.0000
稀释因子: : 1.0000
样品量: : 1.00000 [ng/ul] (校正中没有使用)
内标使用乘积因子和稀释因子

信号 1: DAD1 C, Sig=254,4 Ref=off

峰 #	保留时间 [min]	类型	峰宽 [min]	峰面积 [mAU*s]	峰高 [mAU]	峰面积 %
1	8.148	BV	0.4249	340.52252	12.45824	0.8754
2	8.745	VV	0.5452	1094.35022	29.88571	2.8134
3	11.240	VB	0.8178	3.74630e4	584.44922	96.3112

总量 : 3.88979e4 626.79317

Figure S42. HPLC of **9e**

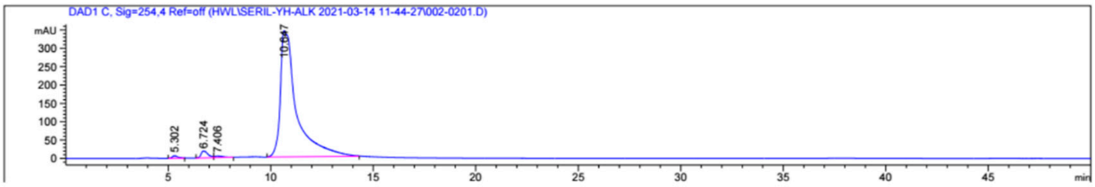

面积百分比报告

排序 : 信号
乘积因子: : 1.0000
稀释因子: : 1.0000
内标使用乘积因子和稀释因子

信号 1: DAD1 C, Sig=254,4 Ref=off

峰 #	保留时间 [min]	类型	峰宽 [min]	峰面积 [mAU*s]	峰高 [mAU]	峰面积 %
1	5.302	BB	0.2914	123.40794	6.44585	0.6288
2	6.724	BV	0.3637	439.23438	18.58435	2.2380
3	7.406	VB	0.3775	117.28642	4.05410	0.5976
4	10.647	BB	0.7029	1.89466e4	343.81015	96.5357

总量 : 1.96265e4 372.89445

Figure S43. HPLC of **9f**

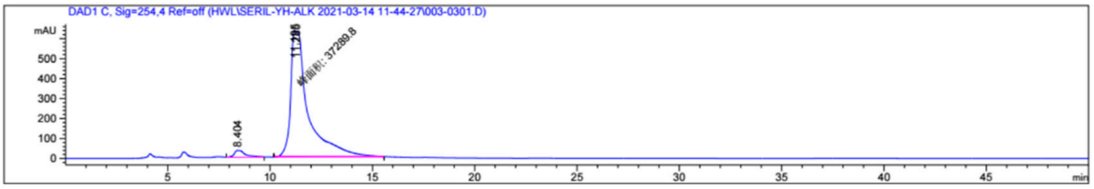

面积百分比报告

排序 : 信号
乘积因子: : 1.0000
稀释因子: : 1.0000
内标使用乘积因子和稀释因子

信号 1: DAD1 C, Sig=254,4 Ref=off

峰 #	保留时间 [min]	类型	峰宽 [min]	峰面积 [mAU*s]	峰高 [mAU]	峰面积 %
1	8.404	VB	0.4924	1279.72363	33.75162	3.2619
2	11.195	MM T	0.9866	3.72898e4	629.91400	95.0495
3	11.285	BB R	0.0140	662.45190	624.65167	1.6885
总量 :				3.92320e4	1288.31730	

Figure S44. HPLC of **9g**

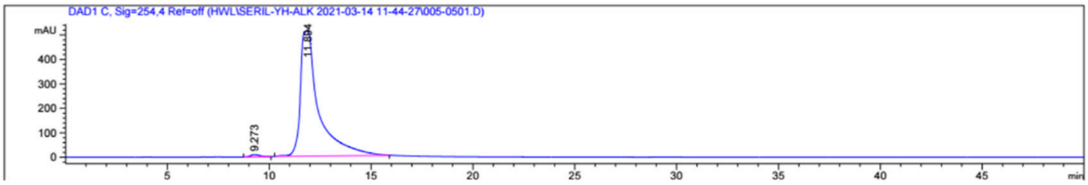

面积百分比报告

排序 : 信号
乘积因子: : 1.0000
稀释因子: : 1.0000
内标使用乘积因子和稀释因子

信号 1: DAD1 C, Sig=254,4 Ref=off

峰 #	保留时间 [min]	类型	峰宽 [min]	峰面积 [mAU*s]	峰高 [mAU]	峰面积 %
1	9.273	BB	0.4897	268.10114	8.50723	0.8230
2	11.894	BB	0.8021	3.23079e4	514.84302	99.1770
总量 :				3.25760e4	523.35025	

Figure S45. HPLC of **9h**

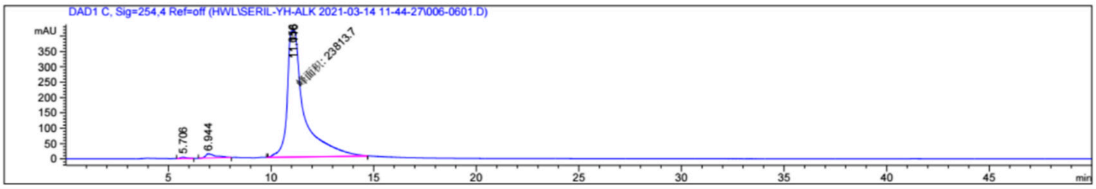

==

面积百分比报告

==

排序 : 信号
乘积因子: : 1.0000
稀释因子: : 1.0000
内标使用乘积因子和稀释因子

信号 1: DAD1 C, Sig=254,4 Ref=off

峰 #	保留时间 [min]	类型	峰宽 [min]	峰面积 [mAU*s]	峰高 [mAU]	峰面积 %
1	5.706	BB	0.2957	84.56963	4.04803	0.3458
2	6.944	BB	0.4534	483.34998	13.90822	1.9764
3	11.056	BB R	2.44e-3	75.03142	411.18445	0.3068
4	11.116	MM T	0.9644	2.38137e4	411.55344	97.3711

总量 : 2.44567e4 840.69414

Figure S46. HPLC of **9i**

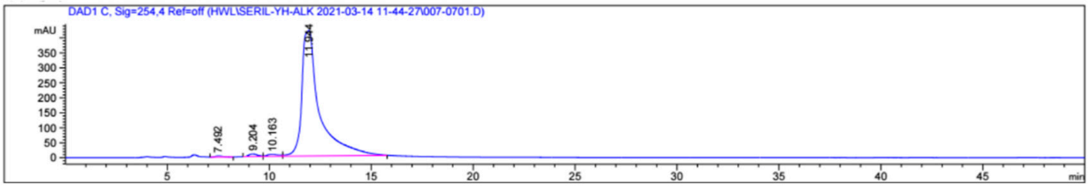

==

面积百分比报告

==

排序 : 信号
乘积因子: : 1.0000
稀释因子: : 1.0000
内标使用乘积因子和稀释因子

信号 1: DAD1 C, Sig=254,4 Ref=off

峰 #	保留时间 [min]	类型	峰宽 [min]	峰面积 [mAU*s]	峰高 [mAU]	峰面积 %
1	7.492	BB	0.3892	89.88037	3.05495	0.3478
2	9.204	BV	0.4751	244.53281	8.21865	0.9463
3	10.163	VV	0.4849	268.47052	6.65210	1.0389
4	11.944	VB	0.7781	2.52382e4	419.37961	97.6670

总量 : 2.58411e4 437.30531

Figure S47. HPLC of **9j**
